# Organic Selenium Quality and Quantity in Soils Are Related to Organic Matter Composition and Driven by Land Use

**DOI:** 10.1021/acs.est.5c15430

**Published:** 2026-03-04

**Authors:** Maja B. Siegenthaler, Lenny H. E. Winkel, Reto G. Meuli, Julie Tolu

**Affiliations:** † 28499 Eawag, Swiss Federal Institute of Aquatic Science and Technology, Department of Water Resources and Drinking Water (W+T), Überlandstrasse 133, Dübendorf 8600, Switzerland; ‡ Department of Environmental Systems Sciences (D-USYS), ETH Zurich, Swiss Federal Institute of Technology, Institute of Biogeochemistry and Pollutant Dynamics (IBP), Group of Inorganic Environmental Geochemistry, Universitätstrasse 16, Zurich 8092, Switzerland; § Agroscope, Agroecology and Environment, Soil Quality and Soil Use, Reckenholzstrasse 191, Zurich 8046, Switzerland

**Keywords:** soils, selenium, sulfur, organic matter degradation, agricultural management, size exclusion chromatography, ICP-MS/MS, pyrolysis-GC/MS

## Abstract

The micronutrient selenium (Se) exhibits a narrow range between essentiality and toxicity. In soils, Se speciation influences its mobility and plant availability, with implications for addressing unsafe Se levels in plant-based nutrition. We investigated how Se speciation varies with the molecular composition of organic matter (OM) in 92 Swiss topsoils spanning different land uses (i.e., croplands, grasslands, and forests). OM composition was characterized using pyrolysis-gas chromatography/mass spectrometry (Py-GC/MS), while Se speciation was determined in NaOH extracts using size exclusion chromatography coupled to UV and elemental mass spectrometry (SEC-UV-ICP-MS/MS). We found that Se speciation strongly relates to OM decomposition status and pH, and drastically differs between soil land uses. Cropland soils exhibited higher proportions of Se oxyanions and small hydrophilic organic Se, whereas forest and grassland soils contained more larger, aromatic organic Se compounds. Overall, these larger Se forms correlated with fresh and/or poorly decomposed, plant-derived OM, while oxyanions and small hydrophilic Se were linked to decomposed OM. Additionally, Se extractability by NaOH decreased with increasing soil pH, which may be due to stronger SOM stabilization, microbial processes, or higher Ca contents at higher pH. These results have important implications for Se plant availability considering land use changes and SOM degradation.

## Graphical abstract

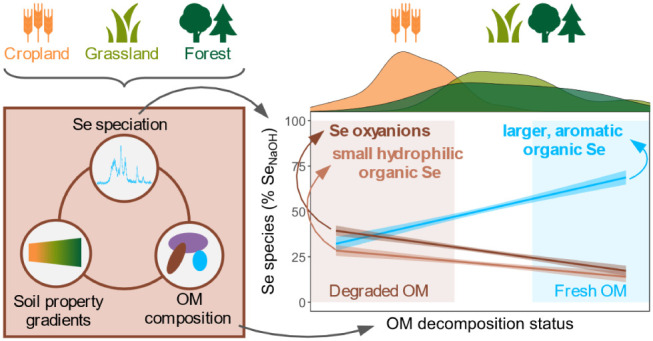

## Introduction

Micronutrient deficiencies remain a prevalent global health concern.^1,2^ Selenium (Se) is one of the essential micronutrients for humans and animals, but its safe intake range is narrow.^3,4^ In plant-based diets, Se intake is related to Se concentrations in soils, which are generally low (0.01–2 mg Se kg^–1^, global mean of 0.32 mg Se kg^–1^).^5,6^ Progressing soil degradation and projected declines in atmospheric Se inputs may further lead to lower Se contents in soils and thus in plants and plant-based nutrition.^6,7^ The uptake of soil Se by plants depends on its chemical speciation. The Se oxyanions selenate (SeO_4_^2–^, + VI) and selenite (SeO_3_^2–^, + IV) are known as the most mobile and plant available forms.^8–10^ They are not only present as free ions in soil solution, but also adsorb to mineral surfaces (e.g., weakly complexed to oxides) or coprecipitate with minerals. In contrast, more reduced inorganic Se species, such as mineral Se­(−II) and elemental Se(0), are generally considered immobile.^9^ In addition, large proportions of soil Se (up to 70–90%) exist in organic forms.^11–15^

Organic Se forms play a crucial role in soil Se retention and plant availability. But the effect of organic matter (OM) on plant Se availability can vary widely. For example, the addition of OM as crop residues or manure has been experimentally shown to reduce plant available Se and plant uptake of added selenate, while water-soluble OM has been positively related to Se uptake by wheat.^16–18^ Although organic Se may remain immobilized in the soil solid phase,^19–22^ it could also be released into the soil solution, e.g., through decomposition and oxidation,^18,23–25^ thereby becoming plant available and thus constituting a long-term reservoir of plant available Se. Besides the plant availability, the origin of soil organic Se remains unclear. Organic Se in soils is likely formed via microbial processes or derived from Se-containing plant material, e.g., as Se-containing biomolecules, similarly to the pathways known for sulfur (S).^11,26^ Microbially produced hydrogen selenide­(−II) could be incorporated into soil organic matter (SOM), forming covalent bonds with soil organic carbon (SOC)^27^ or Se could become part of the SOM pool as Se-metabolites and selenoproteins. Diverse microorganisms can synthesize such compounds,^28^ and several studies have reported the formation of unknown or organic Se species in soils related to bacterial or fungal activity.^29–33^ Alternatively, Se oxyanions could bind to SOM through ternary complexation, e.g., with iron (Fe­(III)),^34^ a mechanism known for arsenic (As).^11,35^ However, recent characterization of organic Se forms across a climate gradient suggests that such complexes are either absent or constitute only a minor fraction of total organic Se.^13^ Overall, the factors controlling the quantity and quality of organic Se in soils require further investigation.^15^

Here, we investigated how Se speciation varies with soil properties, focusing on how SOM molecular composition relates to organic Se quantity and quality. We hypothesized that (organic) Se speciation varies with OM molecular composition, based on previous studies linking organic Se quantity with land use types,^12,13,36^ which are known to exhibit distinct OM composition.^37,38^ We therefore quantified organic Se fractions of different size and/or chemical properties and Se oxyanions in NaOH extracts of a Swiss soil collection, consisting of 92 topsoils from croplands, grasslands, and forests. This soil collection covers wide gradients in soil properties (e.g., pH, 3.2–7.5; SOC, 1–16%) and, is representative of most Se concentrations reported in other countries (<1 mg kg^–1^). Organic Se fractions were quantified together with Se oxyanions and Se associated with mineral nanoparticles using a recently developed method of size exclusion chromatography (SEC) coupled to UV and inductively coupled plasma tandem mass spectrometry (ICP-MS/MS).^13^ SOM molecular composition was determined using pyrolysis-gas chromatography/mass spectrometry (Py-GC/MS), providing semiquantitative data of over 100 pyrolytic organic compounds. These compounds are specific for different OM biochemical classes and are indicative of OM sources and degradation status.^39^ We showed that SOM molecular composition and pH are related to organic Se quantity and quality. More specifically, the distribution of larger and aromatic NaOH-extractable organic Se versus small hydrophilic NaOH-extractable organic Se or Se­(IV) is linked to OM decomposition status.

## Materials and Methods

### Site Selection and Soil Collection

We analyzed topsoils (0–20 cm) from 92 sites in Switzerland (Figure S1), including croplands (n = 30), grasslands (n = 33), and forests (n = 28). These sites are independently managed by farmers and foresters but are collectively part of the Swiss Soil Monitoring Network,^40^ reflecting typical Swiss agroecological zones.^41^ The sites overall covered 18 different soil types according to the Swiss soil classification (Table S1) and spanned gradients in elevation (324–2400 m.a.s.l.), mean annual temperature (MAT, −1.4–11.1 °C), and mean annual precipitation (MAP, 646–2121 mm) (Figure S1). Samples were dried at 40 °C, sieved at 2 mm, and finely ground using a swing mill (MM400, Retsch GmbH) with zirconium oxide grinding balls for 4 min at 25 Hz. The analyses conducted in this study were performed on one archived composite topsoil replicate per site sampled between 2010 and 2017 (Method S1).

### Analytical Methods

#### Bulk Soil Properties

Bulk soil properties, determined using Swiss standard methods (Method S1),^42^ were taken from the Swiss Soil Monitoring Network database (Table S2). The following soil properties were included: Soil pH, soil organic carbon (SOC), total nitrogen (TN), soil texture (clay and silt content), potential cation exchange capacity (CEC_pot_), amorphous iron oxides (Fe_oxa_), basal soil respiration, and apparent density of the fine earth (bulk density, ADFE).

#### Soil Se, S, and Other Element Concentrations

Concentrations of Se, S, iron (Fe), and manganese (Mn) in soils were determined after microwave-assisted digestion (with a mixture of HNO_3_, H_2_O_2_, and HF; Method S2), using an Agilent 8900 ICP-MS/MS equipped with an Elemental Scientific prepFAST autosampler (Method S3 and Table S3). Each soil sample was digested in duplicate, and each digestion batch included three digestion blanks and four certified reference materials (CRMs, Sigma-Aldrich CRM-044 Silt Loam 1, NIST SRM-2709a San Joaquin soil, CNMR GBW 07405 Chinese Yellow-red soil, and CNMR GBW 07408 Chinese Loess). As an additional quality control (QC), one of the soil samples from the soil collection was digested in each batch. In addition, two aqueous CRMs diluted in the corresponding digest matrix were analyzed with the soil digests, i.e., the NIST 1643f (trace elements in freshwater; NIST) and the HPCE Standard Solution 29235 (Multielement Anion in water, Sigma-Aldrich). Results obtained for the QCs are reported in Tables S4–S6. Concentrations of calcium (Ca) and magnesium (Mg) for the same soil samples as digested here were taken from the Geochemical Soil Atlas of Switzerland (Table S2).

#### Se and S Concentrations and Speciation in NaOH Extracts

Se and S speciation were determined in single NaOH extracts, obtained by directly extracting soils with 0.1 M NaOH and a 1:33 soil:solution ratio. NaOH extracts are known to include organic Se and S and mineral-adsorbed Se and S oxyanions.^9,11^ In comparison to sequential extractions, single NaOH extracts contain additionally water-soluble (Se oxyanions and selenoamino acids) and exchangeable (mineral-adsorbed Se), Se otherwise removed in prior extraction steps. The extraction and analyses were conducted using previously developed protocols and are described in Method S4.^13,43^ Four extraction blanks, six replicates of one soil sample from the Swiss soil collection, and three replicates of two soils from the Kohala climate gradient (Hawaii) analyzed by Tolu et al. (2022)^13^ were included as QCs. Total element concentrations in the NaOH extracts were determined by ICP-MS/MS as described above with two aqueous CRMs (NIST 1643f and HPCE Standard Solution 29235) diluted in the corresponding NaOH matrix. Inorganic selenite (SeO_3_^2–^(IV)) and selenate (SeO_4_^2–^(VI)) were quantified by anion exchange chromatography (AEC) using an Agilent 1260 Infinity II high-performance liquid chromatography (HPLC) coupled to the Agilent 8900 ICP-MS/MS. Detection limits (Mean (M) ± standard deviation (SD)) were 9.7 ± 0.5 ng L^–1^ and 3.1 ± 0.2 μg kg^–1^ for Se­(IV), and of 16 ± 4 ng L^–1^ and 5 ± 1 μg kg^–1^ for Se­(VI). Size resolved Se and S speciation was determined by size exclusion chromatography (SEC) using the same Agilent 1260 Infinity II HPLC equipped with an Agilent diode array detector (DAD) and coupled to the Agilent 8900 ICP-MS/MS. Five Se and S SEC fractions were determined, i.e., F1 (organo)­mineral nanoparticles; F2 larger, more negatively charged, Fe-enriched, aromatic OM; F3 smaller, less negatively charged, aromatic OM; F4 small hydrophilic OM; F5 free oxyanions (Figure S3). Results obtained for the QCs are reported in Tables S4, S6, and S7. Furthermore, dissolved organic carbon (DOC) concentrations were measured in 25–100 times diluted NaOH extracts using a TOC analyzer (Shimadzu TOC-L CSH).

#### Organic Matter Molecular Composition

Organic matter (OM) molecular composition was determined on solid samples using a multishot EGA/PY-3030D Pyrolyzer (Frontier Laboratories Ltd.) coupled to a TRACE 1300 GC/ISQ 7000 MS (Thermo Fisher Scientific) as optimized previously (Method S6).^39^ Relative abundances of each identified pyrolytic­(Py)-product were calculated by dividing the area of each Py-product by the sum of peak area of the 150 identified Py-products (Table S9). The peak areas (signal intensity) of identified Py-products are proportional to the concentrations of the organic compounds they derive from, with the sum of identified peak areas correlating with SOC and the sum of identified N compounds correlating with soil TN (Figure S4). Based on nine samples analyzed in triplicates, the reproducibility, expressed as relative standard deviation of the relative peak abundance of all identified Py-products, was 21 ± 12% (M ± SD; minimum (Min)–maximum (Max), 0–35%) but varied between the biochemical classes (Table S10).

### Statistical Analyses

Statistical analysis and data visualization were conducted in R (version 4.4.0, 2024, The R Foundation for Statistical Computing) within R studio (2024.12.0 Build 467, Posti Software, PBc). Differences between land uses were assessed based on estimated marginal means obtained for linear models by *emmeans* version 1.10.5.^44^ Correlations are reported as Spearman rank correlation coefficients (r_S_) with the corresponding holm-adjusted P-values (*psych* version 2.4.6.26).^45^ The variability in OM molecular composition was investigated by principal component analysis (PCA) based on the centered and scaled relative abundances of groups of Py-products (noted Py-groups thereafter). Figures were produced with *ggplot2* version 3.5.1.^46^ When plotting one variable as the function of another, we report the adjusted coefficients of determination (R^2^_adj_), P-values (P), and number of samples (n) of the linear models fitted using *ggpmisc* version 0.6.0.^47^ More detailed information can be found in Method S7.

## Results and Discussion

### Bulk Properties and Concentrations of Se and S in Swiss Soils

The soil collection spanned wide gradients in SOC content (1.2–16.2%), TN content (0.1–1.1%), and pH (3.2–7.5) across and within land use types (Table 1). On average, SOC was significantly lower in cropland than forest and grassland, while TN was lower in cropland and forest than in grassland, resulting in lower C/N in cropland and grassland than forest. Exceptionally high SOC and TN were observed for three converted peat soils (two croplands and one grassland; Figure S5). In contrast to SOC and TN, pH was significantly higher for cropland than grassland and forest. These findings follow expected land use patterns.^48–50^

**Table 1:** SOC, TN, C/N, pH, and Total Se and S Concentrations (Se_Soil_ and S_Soil_) in the Swiss Soil Collection^a^

Parameter	Unit	Land use	M	SD	Mdn	Min	Max	n
SOC	%	Cropland	2.9^a^	3.1	2.0	1.2	16.2	31
		Grassland	5.1^b^	1.9	4.6	3.0	12.5	33
		Forest	5.8^b^	3.4	4.9	2.4	15.6	28
TN	%	Cropland	0.28^a^	0.21	0.22	0.11	1.13	31
		Grassland	0.47^c^	0.12	0.47	0.31	0.81	33
		Forest	0.35^b^	0.17	0.27	0.17	0.72	28
C/N	ratio	Cropland	9.6^a^	1.5	9.5	7.5	14.4	31
		Grassland	10.3^a^	1.6	9.8	8.0	13.7	33
		Forest	16.7^b^	4.1	15.9	10.7	27.1	28
pH		Cropland	6.3^b^	0.8	6.1	4.9	7.5	31
		Grassland	5.2^a^	0.7	5.2	3.8	6.3	33
		Forest	4.8^a^	1.2	4.5	3.2	7.5	28
Se_Soil_	μg Se kg^–1^	Cropland	305	223	203	103	1089	31
		Grassland	331	151	313	96	753	33
		Forest	358	237	259	87	916	28
S_Soil_	mg S kg^–1^	Cropland	475^a^	415	343	189	2302	31
		Grassland	653^b^	184	653	386	1272	33
		Forest	449^a^	254	343	175	945	28

a Mean (M), standard deviation (SD), median (Mdn), minimum (Min), and maximum (Max) of n samples per land use are given. Superscript letters next to the mean denote significant differences between land uses (*P* ≤ 0.05) for each parameter based on estimated marginal means. The three converted peat soils were removed as outliers prior to the statistical comparison of land uses for SOC, TN, pH, and S_Soil_.

Soil Se concentrations (Se_Soil_) were low (M ± SD), 330 ± 204 μg kg^–1^; median (Mdn), 263 μg kg^–1^), as reported globally (M, 320 μg kg^–1^) and for Europe (Mdn, 350 μg kg^–1^).^6,51^ They spanned a wide range (87–1089 μg kg^–1^), and no differences between land uses were observed (Table 1). Soil S concentrations (S_Soil_) also spanned a wide range (175–2302 mg kg^–1^; M ± SD, 531 ± 310 mg kg^–1^; Mdn, 475 mg kg^–1^) but were higher than those reported previously for Swiss soils (n, 1201; Mdn, 350 mg kg^–1^),^50^ cropland soils (n, 2108; Mdn, 207 mg kg^–1^) and grassland soils (n, 2024; M, 295 mg kg^–1^) across Europe.^51,52^ However, previous studies employed aqua regia for digesting the samples, while we used HF with HNO_3_ and H_2_O_2_, leading to full soil S recovery (Table S5). In contrast to Se_Soil_, S_Soil_ was significantly larger in grassland soils than forest and cropland soils, as also reported for the larger Swiss data set.^50^

In line with previous studies,^13,15,36,53–56^ Se_Soil_ correlated positively with CEC_pot_ (r_S_ = 0.74), TN (r_S_ = 0.70), S_Soil_ (r_S_ = 0.69), clay (r_S_ = 0.58), SOC (r_S_ = 0.53), and Fe_Soil_ (r_S_ = 0.55) (*P* < 0.001; Table S11). S_Soil_ correlated positively with TN (r_S_ = 0.91), SOC (r_S_ = 0.74), CEC_pot_ (r_S_ = 0.77), Se_Soil_ (r_S_ = 0.69), and Fe_oxa_ (r_S_ = 0.57) (*P* < 0.001; Table S11), as previously observed.^50^

### Se and S Speciation in NaOH Soil Extracts Differ with Land Use Types

NaOH extracts of Swiss soils contained 1.2–22.7 μg Se L^–1^, accounting for 28–101% (M ± SD, 61 ± 17%) of Se_Soil_ in line with previously reported extraction efficiencies.^12,13,15,18,26,36,43,57–61^ Smaller proportions of S_soil_ (15–70%; M ± SD, 43 ± 13%) and SOC (11–58%; M ± SD, 38 ± 11%) than for Se_Soil_ were extracted by NaOH, as previously reported,^13,36^ and the Se, S and SOC extractabilities correlated strongly with each other (Figure S6).

With SEC-UV-ICP-MS/MS, we obtained a full recovery of the Se species in all samples (M ± SD, 98 ± 7%), and free Se­(IV) concentrations matched those determined by AEC-ICP-MS/MS (Figure S7). Note that Se­(VI) was mostly below the detection limit or not quantifiable. The free Se­(IV) in NaOH extracts was either originally present in the soil solution or adsorbed to mineral surfaces.^13^ We did not find any Se associated with the fraction F1 “(organo)­mineral nanoparticles”, that likely includes Fe (oxy)­hydroxides nanoparticles, which are mobilized from soils and/or formed during NaOH extraction.^62,63^ In line with this observation, no Se was found in the “(organo)­mineral nanoparticles” fraction for the 25 volcanic soils from the Kohala climate gradient (Hawaii),^13^ i.e., the first study using the same SEC-UV-ICP-MS/MS method to determine Se speciation in soil NaOH extracts. In combination with the full Se species recovery, our result, which covers 92 soils from different land uses, demonstrates that Se does not commonly exist in association with mineral nanoparticles and colloids in NaOH soil extracts. The peaks of the first organic fraction F2 “larger, more negatively charged, Fe-enriched, aromatic OM”, where Fe­(III) is likely complexed with OM,^13,64^ elute closely to the peaks of F3 “smaller, less negatively charged, aromatic OM”. Deconvoluting (i.e., resolving the underlying individual peaks that overlap) fractions F2 and F3 reproducibly was more difficult for the Swiss soils than for the Hawaii soils in the previous study,^13^ due to five times lower median Se concentrations. Indeed, F2 appears only as a small shoulder on the larger F3 peak, and its deconvolution is unstable (even on the same chromatogram with the same deconvolution settings) because small changes in initialization or bounds yield different fits when a weak shoulder is embedded in a dominant peak. Thus, for the Swiss soil sample extracted and analyzed six times, Se concentrations in F2 showed a relative standard deviation of 39% (Table S6). Therefore, F2 and F3 fractions were pooled into one fraction for Se and S, named F2+F3 “larger and aromatic organic Se or S”, which is thereafter shown alongside fractions F4 “small hydrophilic organic Se or S”, and F5 “Se or S oxyanions” (Figure 1).

Both Se and S were more associated with the organic fractions (F2+F3+F4, 72 ± 7% of Se_NaOH_ and 76 ± 6% of S_NaOH_, respectively) than present as oxyanions (F5, 28 ± 7% Se_NaOH_ and 24 ± 6% S_NaOH_, Figure 1). However, while organic S mainly occurred as larger and aromatic organic S (72 ± 6% S_NaOH_ in F2+F3), organic Se had an important fraction of small hydrophilic compounds (21 ± 7% Se_NaOH_ in F4 and 50 ± 11% Se_NaOH_ in F2+F3), which is quantitatively unimportant for S (4 ± 2% S_NaOH_). A very similar pattern was observed for the Hawaii soils, in which 56 ± 14% of Se_NaOH_ and 78 ± 19% of S_NaOH_ were found in F2+F3 versus 19 ± 7% and 4 ± 2% in F4.^13^ Together, these findings from Swiss and Hawaii soils suggest that small hydrophilic organic Se compounds may generally be an important Se fraction in soils.

**Figure 1:**
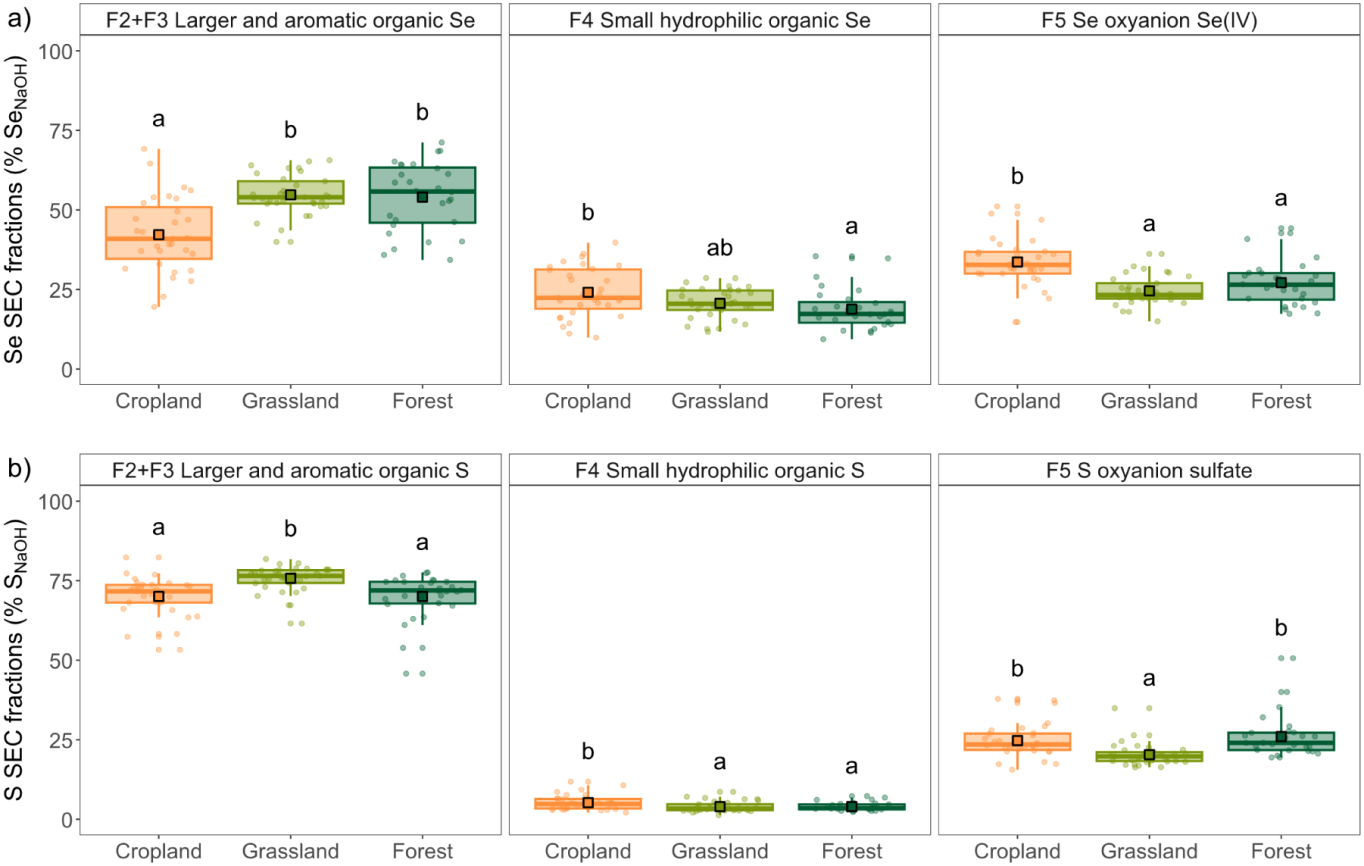
Se and S speciation in NaOH extracts of Swiss soils from different land use types determined by SEC-UV-ICP-MS/MS. The data shows the proportions of Se and S in detected SEC fractions F2+F3 “Larger and aromatic organic Se or S”, F4 “Small hydrophilic organic Se or S”, and F5 “Se or S oxyanions” in % of total Se and S in NaOH extracts (i.e., Se_NaOH_ and S_NaOH_). The boxplots show the interquartile range (IQR) as box, representing the middle 50% of the data between the 25th and 75th percentiles, the median marked by the middle line, and the whiskers extending to 1.5 times the IQR. The mean is shown as a square. The points represent the individual sites. All elements are colored according to land use (cropland, grassland, forest). Different letters denote significant differences between land uses (*P* ≤ 0.05) per fraction based on estimated marginal means. The three converted peat soils were removed as outliers prior to the statistical comparison of land uses.

Both Se_NaOH_ and S_NaOH_ speciation significantly differed between land uses, with more pronounced differences observed for Se than S (Figure 1). The proportion of Se oxyanions (F5) was significantly larger in cropland (M ± SD, 34 ± 7%) than forest (27 ± 7%) and grassland (25 ± 5%) soils, as previously reported for French soils.^36^ Higher proportions of small hydrophilic organic Se (F4) were also found for croplands (24 ± 8%) compared to forests (19 ± 6%). Complementary, the proportion of Se_NaOH_ present in the larger and aromatic organic fractions F2+F3 was significantly lower in croplands (42 ± 13%) than in forests (54 ± 11%) and grasslands (55 ± 6%). In contrast to Se oxyanions, the proportion of S_NaOH_ present as sulfate (F5) was significantly larger in cropland (25 ± 5%) and forest (26 ± 7%) than grassland (20 ± 4%) soils, while the contribution of F2+F3 was significantly larger for grassland soils (76 ± 6%; Figure 1). However, similarly to Se, the proportion of small hydrophilic organic S (F4) was largest in cropland soils (5 ± 3%). The differences in the proportions of Se fractions in NaOH extracts between land uses are mainly driven by the larger absolute differences in Se concentrations in the largest fraction of organic Se (F2+F3) (Figure S8). Generally, our results show that larger and aromatic organic Se and small hydrophilic organic Se are important forms of Se in NaOH soil extracts, it remains to be elucidated how the SOM composition is related to these organic Se fractions. The pronounced differences in (organic) Se speciation between land use types, i.e., the higher proportions of Se oxyanions (F5) and small hydrophilic organic Se (F4) in cropland soils and higher proportions of larger, aromatic organic Se (F2+F3) in forest and grassland soils, suggest a connection with SOM quality, known to vary with land use.^37,38^ We hypothesize that the organic Se fractions determined here are related to SOM composition, which is driven by OM sources and decomposition processes.

### Variability in Organic Matter Molecular Composition across and within Land Use

To further interpret the differences in Se and S speciation across and within land use types, we investigated the variability in OM molecular composition in the 92 Swiss soils. To this effect, we applied principal component analysis (PCA) to the OM data set (Figure 2). This data set includes 150 pyrolytic Py-products belonging to 13 biochemical OM classes (e.g., carbohydrates, N compounds, chitin-derived Py-products, phenols, lignin, chlorophyll, *n-*alkenes, *n-*alkanes, alkan-2-ones, steroids, carboxylic acids, S compounds and (poly)­aromatics), which we grouped into 41 groups based on similarities in the molecular structure and origin of the Py-products (Tables S9 and S12).^65,66^

Positive loadings on PC1, which covers 32% of total variance, were found for (i) anhydrosugars (e.g., levoglucosan, anhydropentose) and (alkyl)­pyranones that are Py-products of high-molecular-weight carbohydrates and cellulose;^67–69^ (ii) phytadienes that are Py-product of chlorophyll;^70^ (iii) diketopiperazines and (alkyl)­pyrazines that are Py-products of amino acids and proteins;^71^ (iv) carboxylic acids (chain-lengths between 14 and 18 C; carboxylic acids C14–16 and C18); and (v) steroids (Figure 2a). These Py-groups and, more generally, polysaccharides, amino acids, proteins, chlorophyll, carboxylic acids, and steroids, are well-known to be rapidly degraded with OM decomposition and microbial processing.^67,70,72,73^ In contrast, we found negative PC1 loadings for degradation products of carbohydrates (e.g., (alkyl)­furans (alkyl)­furanones) and of proteins, amino acids and/or chlorophylls (e.g., (alkyl)­pyrroles, aromatic N, (alkyl)­pyridines, diketodipyrroles, (alkyl)­phenols, (alkyl)­indoles).^67,74–76^ Negative PC1 loadings were also observed for polyaromatics, which are known to increase with OM degradation.^67,75,77^ A similar gradient in OM degradation status, ranging from fresh OM in roots and organic soil horizons (e.g., levoglucosan an anhydrosugar, 3-hydroxy-2-methyl-(4H)-pyran-4-one belonging to (alkyl)­pyranones) to decomposed OM in deeper soil horizons (e.g., pyrrole and indole as degradation products of proteins, pyrene a polyaromatic compound) was observed in forest soils using Py-GC/MS combined with NMDS (i.e., a statistical approach similar to PCA).^78^ We thus interpret PC1 as separating samples enriched in fresh and/or poorly decomposed OM (positive loadings) from samples richer in compounds originating from OM decomposition (negative loadings). This interpretation does not imply that all Py-products plotting on PC1’s negative side originate exclusively from OM decomposition, nor that all compounds on the positive side represent strictly “fresh” and rapidly degradable material (Table S12). For example, polyaromatic compounds do not only derive from OM degradation but may also directly originate from plants and microbes, either because these organisms naturally contain polyaromatic structures or because their (wax) lipids partially form polyaromatic compounds during pyrolysis. Importantly, it is the coherent grouping of the different Py-compound groups in the PCA space rather than any single marker alone that supports our interpretation that PC1 reflects OM decomposition status.

The enrichment of soils in fresh and/or poorly decomposed OM (which plots on PC1’s positive side) may result from different, and likely overlapping, processes. These include (i) (substantial) inputs of fresh plant OM by vegetation and/or (ii) the accumulation of labile plant and microbial OM compounds (e.g., cellulose, proteins, chlorophyll) due to physical protection through mineral associations and/or slower decomposition. The positive correlations found between PC1 and SOC, C/N, and basal soil respiration (measured under optimal temperature and soil moisture conditions; Figure 2a) support the interpretation that PC1’s positive side reflects inputs and accumulation of nondecomposed plant OM. The negative correlations between PC1 and MAT as well as pH (Figure 2a) further indicate slower decomposition of fresh plant OM under colder and more acidic conditions. Consistently, cellulose degradation rates increase with increasing soil temperature but decrease with decreasing pH in forest soils,^79,80^ while liming enhances crop residue decomposition in agricultural soils.^81^ Moreover, lower MAT and pH are globally associated with higher particulate OM concentrations in topsoils, which has been attributed to slower decomposition of fresh OM under acidic and colder conditions.^82^

We found that cropland soil, except for one converted peat soil, have negative PC1 scores and are thus characterized by decomposed OM, while grasslands and forests span from negative to positive PC1 scores with the largest span for forests (Figure 2b). For example, anhydrosugars (Py-products of fresh high-molecular-weight carbohydrates and cellulose; positive PC1-loadings) were more abundant in grassland (Mdn, 8.9%) and forest (6.3%) than in cropland (3.6%) soils (Figure S9). In contrast, for example, (alkyl)­pyridines (degradation products of proteins and chlorophyll; negative PC1-loadings) were more abundant in cropland (Mdn, 2.3%) than in grassland (1.1%) and forest (0.7%) soils. The large PC1 span of grasslands is likely influenced by some sites being located at higher altitudes and thus experiencing lower MAT (Figure S1), which can slow down decomposition.^82^ The distribution of our sites along PC1 from fresh and/or poorly decomposed OM to degraded OM is in line with particulate OM being most sensitive to land use change,^83^ and the proportion of SOM present as particulate OM tending to decrease while mineral-associated OM tending to increase in the order of forests, grasslands, croplands.^82,84^

**Figure 2:**
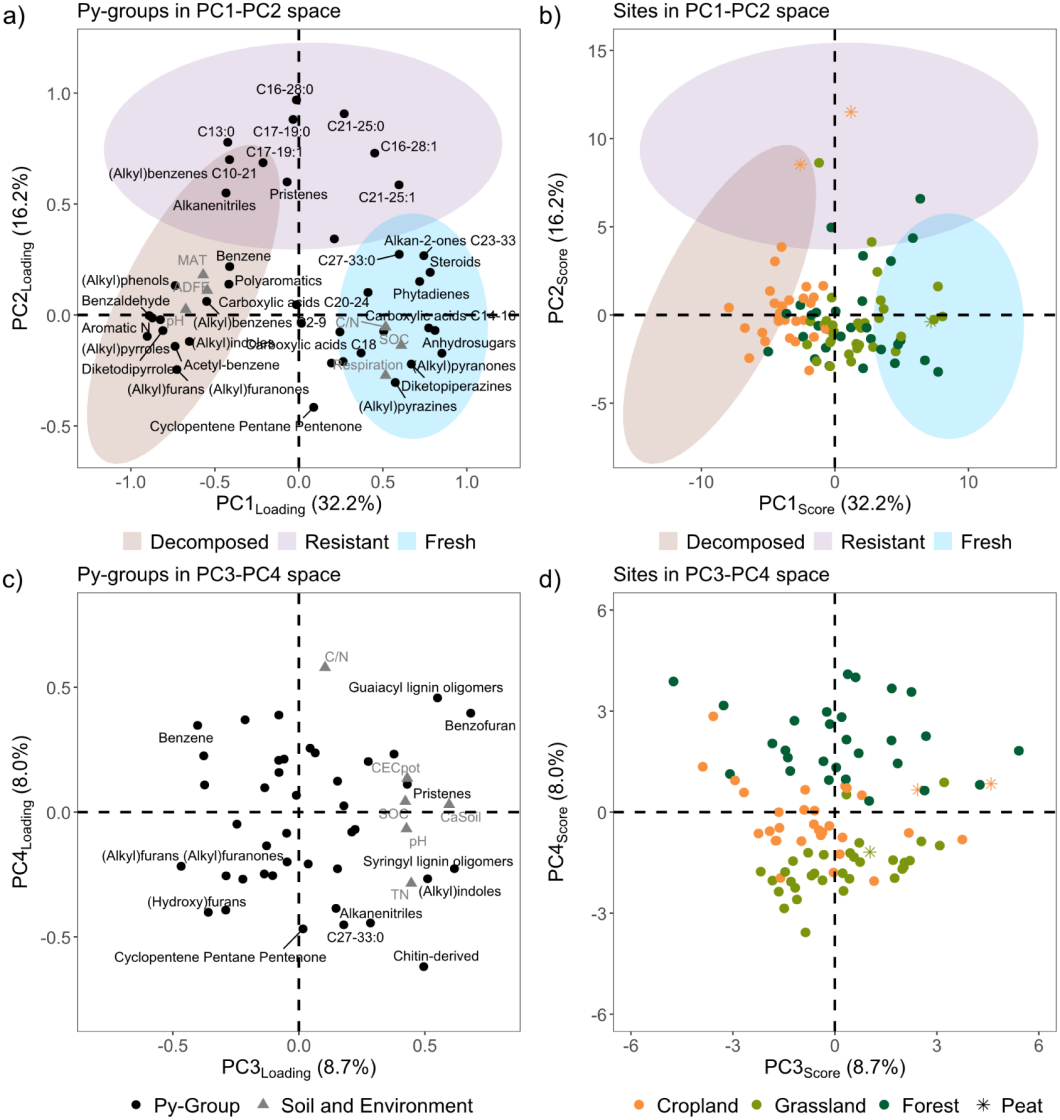
Variability in OM molecular composition across Swiss soils developed on distinct land use highlighted by principal component analysis (PCA) performed using the Py-GC/MS data set. Panels a) and c) show the loadings of the 41 identified Py-groups (active variables; black dots) for the four extracted principal components, which capture together 65% of total variance. Loadings of soil properties and environmental parameters (passive variables; gray triangles) are shown when these variables correlated significantly (r_S_ > 0.4 and *P* < 0.001) with one of the respective PCs. The colored ellipses indicate the decomposition status (resistant, decomposed, fresh and/or poorly decomposed) according to the categorization of the Py-groups shown in Table S12. Panels b) and d) show the scores of the soil samples, which are colored according to land use (cropland, grassland, forest).

On the positive side of PC2 (16% of total variance), we found short to midchain *n*-alkanes (C16–28:0, C21–25:0, C17–19:0, C13:0), *n*-alkenes (C16–28:1, C17–19:1, C21–25:1), and alkanenitriles (Figure 2a), which are Py-products of cell wall and wax lipids and of biomacromolecules, well-known for their resistance to degradation compared to carbohydrates, proteins, and chlorophyll.^77,85–87^ We also found pristenes, which indicate resistant degradation products of chlorophylls.^70^ No Py-products plotted significantly on PC2’s negative side and PC2 did not correlate to any bulk soil properties. We interpret PC2’s positive side to represent organic compounds resistant to decomposition. High PC2 scores were, however, only obtained for the two cropland converted peat soils (Figure 2b). The fact that the converted peat soils plot on PC2’s positive side matched with previous studies showing the accumulation of aliphatic compounds in peat during anaerobic decay,^69,75,88,89^ and indicates the uniqueness of these two converted peat soils within the study soil collection.

PC3 (9% of total variance) showed positive loadings for lignin compounds (guaiacyl-and syringyl-units) that are specific for vascular plants^90^ together with (alkyl)­indoles, pristenes, and benzofuran that are Py- and/or degradation products of proteins, chlorophyll and carbohydrates (Figure 2c). In contrast, negative PC3 loadings were observed for degradation products of carbohydrates (i.e., (alkyl)­furans, (alkyl)­furanones)^75,76^ and benzene indicating degraded OM. PC3 correlated most strongly with Ca_Soil_, followed by weaker positive correlations with TN (matching positive loadings for (alkyl)­indoles, that are N compounds), pH, CEC_pot_, and SOC. PC3 may reflect stabilization of SOM by Ca, e.g., Ca^2+^ bridging to mineral surfaces like clay, or Ca-mediated aggregation.^91^ The Ca originates from the carbonate parent material or anthropogenic inputs e.g., liming. Liming and manure application can both stimulate the SOC incorporation into organo-mineral fractions.^92,93^ Even though Ca-related SOM stabilization is generally more relevant at higher pH,^91^ Ca and SOM are also associated in neutral and acidic soils.^94,95^ PC3 is thus mainly linked to intrinsic soil properties, likely related to bedrock material and potentially soil liming (for cropland soils), resulting in gradients along PC3 for all land uses (Figure 2d).

PC4 (8% of total variance) separated guaiacyl lignin oligomers that are more abundant in gymnosperms, e.g., coniferous trees, on the positive side, from syringyl lignin oligomers that are more abundant in angiosperms, on the negative side^96^ (Figure 2c). PC4’s negative side included almost all identified N compounds, i.e., chitin-derived compounds, pyrolytic and degradation products of proteins and amino acids (e.g., diketopiperazines, (alkyl)­pyrazines) as well as alkanenitriles. Correspondingly, PC4 correlated positively with C/N. While all grassland soils had negative PC4 scores, all forest soils had positive PC4 scores (Figure 2d), and PC4 thus represents differences in the soil OM composition driven by differences in the OM compounds produced by different functional plant groups. Legumes (e.g., clover species) are present in grasslands and have high plant N contents (compared to grasses, nonleguminous forbs, and tree leaves),^97,98^ particularly in the form of proteins, due to the fixation of atmospheric N_2_ by their symbiotic rhizobial bacteria.^98,99^ A part of the fixed N gets deposited in the soil via root turnover^100^ and thus contributes to the observed soil N compounds in the form of proteins and chitin.^101,102^

In summary, as expected, the studied soil collection displayed strong variation in SOM molecular composition, predominantly related to OM decomposition status (PC1 and PC2). Positive PC1 loadings represent fresh and/or poorly decomposed OM, while negative ones indicate compounds originating from OM decomposition. PC2’s positive side represents organic compounds resistant to decomposition. Variation in SOM composition is further related to SOM stabilization by Ca (positive PC3 loadings) and OM inputs by different functional plant groups (PC4). We further use the information derived from this PCA done with the OM data set to evaluate how OM molecular composition is linked to Se and S speciation.

### Se and S Speciation Is Linked to Organic Matter Molecular Composition

The proportion of larger and aromatic organic Se (F2+F3) in NaOH extracts correlated positively with PC1 when considering all soils (F2+F3, r_S_ = 0.72, *P* < 0.0001; Table S13; Figure 3a) but also when considering each land use separately (Figure S10), meaning that this organic Se fraction is related to fresh and/or poorly decomposed, plant-derived OM. In contrast, the proportion of Se­(IV) (F5), and to a lesser extent of small hydrophilic organic Se (F4) correlated negatively with PC1, meaning that these Se species are related to decomposed OM (F5, r_S_ = −0.69, *P* < 0.0001; F4, r_S_ = −0.45, P = 0.0002; Table S13; Figure 3a). The Se fractions were correlated to bulk properties linked to PC1, i.e., pH, ADFE (bulk density), SOC, C/N (Table S13, Figures S11 and 2a), but not to PC2–4, thus demonstrating that soil Se speciation differs with OM decomposition status.

The decline of Se_NaOH_ in F2+F3 with OM decomposition, and the associated increase in Se_NaOH_ in Se­(IV) (F5) and F4 suggests that the latter could be decomposition products of the larger organic Se forms found in F2+F3. In line with this interpretation, studies on the decomposition of natural OM showed that larger, more negatively charged and hydrophobic OM fractions produce small and more hydrophilic organic compounds.^103,104^ Also, during the mineralization of organic S, C-bonded S (mainly proteins) is converted to ester-S (oxidized fractions) and then sulfate.^105,106^ Similarly, mineralization of organic Se to Se oxyanions was observed in studies adding selected Se-oxidizing bacteria and fungi to soil.^25,107–110^ Finally, the negative correlations of MAT and pH with PC1 (MAT, r_S_ = −0.57, *P* < 0.0001; pH, r_S_ = −0.67, *P* < 0.0001; Figure 2a) and Se_NaOH_ in F2+F3 (MAT, r_S_ = −0.48, *P* < 0.0001; pH, r_S_ = −0.65, *P* < 0.0001; Table S13) indicate that OM decomposition as well as organic Se mineralization and oxidation is less pronounced in acidic and colder conditions. In line with these observed relationships, a recent lab study showed that oxidation of seleno-methionine and elemental Se to Se­(IV) by Se-oxidizing bacteria added to sterilized soils were higher in alkaline than acidic soils.^110^ Organic Se in F2+F3 itself is possibly formed in soils from Se oxyanions through either assimilatory or dissimilatory reduction by bacteria and/or fungi.^26,107,111,112^ Assimilatory reduction leads to the formation of Se-metabolites and/or selenoproteins. Dissimilatory reduction of Se is known to form hydrogen selenides (HSe^–^) that can interact with OM via electrophilic attacks, thus forming organo-Se compounds. HSe^–^ may also reoxidize into elemental Se(0) nanoparticles, which can interact with proteins, polysaccharides, and lipids, resulting in Se-containing biopolymers.^111,113,114^ Reduction of Se is likely facilitated in soils and sediments enriched in fresh OM.^11,115^ Organic Se formation has indeed previously been related to microbial processes.^29–33^ For example, Vermeiren et al. (2025) observed an increase in soil immobilization of added selenate with increasing microbial activity, as well as high recovery of added selenate in NaOH extracts, which represents an estimation of formed organic Se quantity.^30^ Furthermore, plant-derived organic Se (e.g., selenoamino acids and selenosugars)^116,117^ could directly contribute to organic Se in soil, as known for organic S.^13^ Future studies are needed to disentangle microbial-derived from plant-derived organic Se recovered in SEC fraction F2+F3 and more generally in soil and to investigate whether Se­(IV) and the organic Se recovered in SEC fraction F4 are decomposition products of the larger organic Se forms recovered in F2+F3.

The proportions of S_NaOH_ fractions followed a similar trend along PC1, but the correlations were weaker than for Se (F2+F3, r_S_ = 0.40, P = 0.0042; F4, r_S_ = −0.35, P = 0.0333; F5, r_S_ = −0.33, P = 0.0614; Table S13; Figure 3b), suggesting a less important link between soil S speciation and OM decomposition status than for Se. In contrast to the Se fractions that were not correlated to PC4 of the OM data set, larger and aromatic organic S (F2+F3) correlated negatively and sulfate positively with PC4, which represents differences in soil OM composition driven by functional plant groups (F2+F3, r_S_ = −0.40, P = 0.0047; F5, r_S_ = 0.43, P = 0.0012; Table S13). More precisely, significantly higher proportions of larger and aromatic organic S and lower proportions of sulfate were observed in grasslands (Figure 1). The legumes associated with rhizobial bacteria present in grasslands have high demands for sulfate to fix N_2_ ^118^ and consequently form many proteins (rich in S), which may later contribute to the soil organic S pool. Our data indicates a link between organic S quality (but not Se quality) and soil OM composition driven by functional plant groups, which aligns with the well-known important contribution of plant-derived organic S to the soil organic S pool.^13^

**Figure 3:**
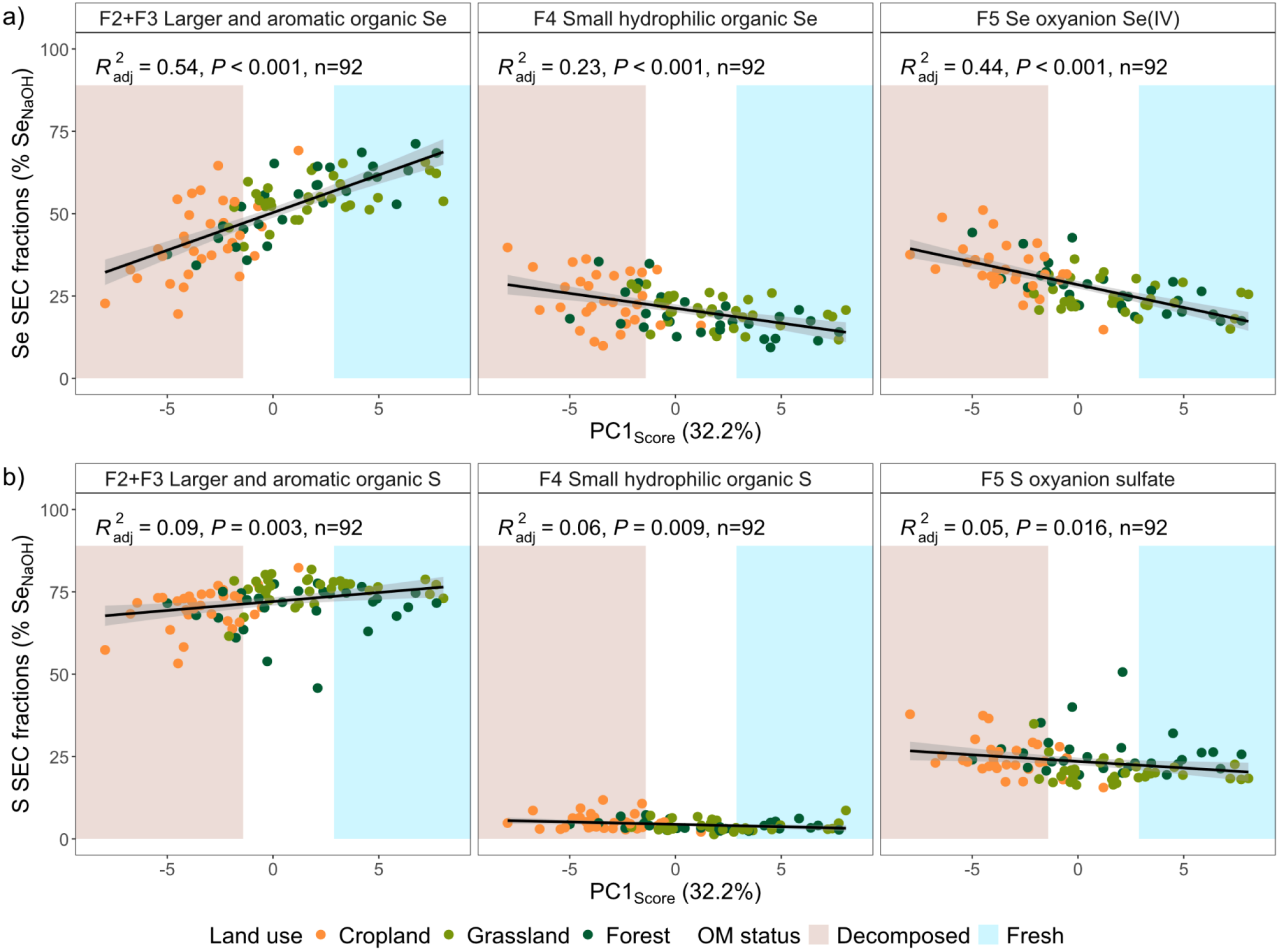
a) Se and b) S fractions (F2+F3, F4, F5) as proportions in NaOH extracts as functions of OM PC1 scores derived from the PCA based on relative abundances of Py-groups. The predicted lines from the linear model fit are shown in black and their 0.95 confidence intervals in gray. The adjusted coefficients of determination (R^2^_adj_), P-values (P), and number of samples (n) of the fitted linear models are reported. The background color indicates the OM decomposition status along PC1 (decomposed versus fresh and/or poorly decomposed). The points represent the individual sites and are colored according to land use (cropland, grassland, forest).

Overall, our data sets reveal a strong link between Se speciation and OM decomposition status across land use types. When fresh and/or poorly decomposed plant-derived OM was abundant (e.g., in forests and grasslands with slower OM decomposition), more NaOH-extractable Se was present as larger and aromatic organic Se (F2+F3), while decomposed OM was related to higher proportions of small hydrophilic organic Se (F4) and Se­(IV), possibly originating from the decomposition of larger and aromatic organic Se. Tolu et al. (2022) showed, by calculating enrichment factors related to the parent rock material for NaOH-extracted Se species in Hawaii soils, that more Se is lost through leaching of Se­(IV) and small hydrophilic organic Se than larger and aromatic organic Se.^13^ The NaOH-extractable Se­(IV) and small hydrophilic organic Se (F4) are thus more mobile than the larger aromatic organic Se in F2+F3. However, while Se­(IV) present in the soil solution is directly plant available, NaOH-extractable Se­(IV) includes Se­(IV) that is adsorbed to mineral surfaces and thus less plant available.^111^ In Tolu et al. (2022), plant Se was positively correlated to Se oxyanions (F5) but negatively to small hydrophilic organic Se (F4) in water extracts.^13^ The small organic Se in F4 might thus need to undergo mineralization to become plant available, even if they were already present in the soil solution.^9,16,18^

The larger and aromatic organic Se in F2+F3 represents a reservoir of organic Se that is more strongly retained in soil but has the potential to become mobile and plant available upon decomposition. When organic Se in F2+F3 gets decomposed, their presumed decomposition products, Se­(IV) and small hydrophilic organic Se in F4, are either adsorbed to mineral surfaces, microbially transformed, taken up by plants, or lost from the soil through leaching. We thus risk a decrease in organic Se along with the decrease in SOC content.^119^ Agricultural management practices, particularly increased carbon inputs from plants or organic amendments like manure, can counteract such SOC loss.^120,121^ In case organic Se formation is facilitated by fresh and/or poorly decomposed plant-derived OM, these inputs may also counteract Se loss by retaining Se as larger and aromatic organic Se (F2+F3). In line with this hypothesis, Laberge-Carignan et al. (2025) showed that more Se was sequestered in sediments enriched with fresh, labile OM than in those enriched with aged, recalcitrant OM during a flow-through reactor experiment fed with low concentrations of Se oxyanions and lake water.^115^ Considering decreasing atmospheric Se inputs,^7^ it will become even more important to understand the link between agricultural management practices and Se speciation and mobility in regards to plant availability, leaching, and retention in soil.

### Organic Se Retention in Soil Is Related to pH

To capture overall Se retention in soil, we also investigated the variability and correlation with soil properties of the Se SEC fractions calculated in relation to total soil Se concentrations (i.e., expressed in % Se_Soil_). This allows us to include the variability in Se extractability by NaOH between soils in our assessment of Se retention. Indeed, the majority of Se_Soil_ (M ± SD, 61 ± 17%) was accounted for in NaOH extracts, but still a large proportion of Se_Soil_ remained nonextractable by NaOH (M ± SD, 39 ± 17%; Figure S12). This nonextractable proportion is subsequently referred to as residual Se (and correspondingly residual S). Residual Se could represent Se associated with stabilized SOM, Se bound to minerals, or elemental Se(0).^9^ Together, residual Se and larger and aromatic organic Se (F2+F3) represent Se more strongly retained in soil. Residual Se correlated strongly positively with pH, strongly negatively with PC1 of the OM data set and Ca_Soil_, and additionally positively with Mn_Soil_, and Fe_Soil_. In contrast, the Se in F2+F3 in soil (% Se_Soil_) correlated strongly positively with PC1 of the OM data set, strongly negatively with pH, and additionally negatively with Ca_Soil_, Mn_Soil_, and clay content (Table S14). Total organic Se (SEC fractions F2+F3+F4) in soil followed the same patterns as its main contributor, Se (F2+F3). Similar correlations with PC1 of the OM data set, pH, and Ca_Soil_ were observed for the respective S fractions. These patterns imply that Se retention as organic Se in F2+F3 is particularly relevant in acidic conditions and when fresh and/or poorly decomposed plant-derived OM is abundant, while residual Se dominates in neutral to alkaline soils and when OM is rather decomposed.

While OM decomposition status influences Se retention, OM quantity estimated by SOC appears less relevant with respect to Se retention in our study. Organic Se proportion in soil (% Se_Soil_) was previously shown to be positively correlated to SOC content,^12,65^ which raised the question of whether Se speciation or even Se plant availability could generally be predicted based on SOC content.^13^ However, we found no simple correlation between the organic Se in soil (F2+F3+F4 in % Se_Soil_) and SOC content in the analyzed Swiss soils (Figure S13). Similarly, organic S was suggested to only accumulate in soils if there is a parallel input of carbon, such as manure or plant material.^122^ For the subset of cropland and grassland sites with management information (n = 33),^40,41^ manure input (as mean annual dry matter application) correlated positively with total Se and S in soil (Se, r_S_ = 0.45, P = 0.0008; S, r_S_ = 0.58, P = 0.0004) but not with the proportions of NaOH-extractable organic Se and S in soil (Se, r_S_ = 0.12, P = 0.51; S, r_S_ = 0.23, P = 0.2033). While manure input tends to increase Se and S accumulation in soil, Se and S speciation remain unaffected. This could be explained by the rapid mineralization of Se and S compounds, such as amino acids, added with manure,^105,124^ or by variable (trace) element and OM quantity and quality across manure types.^125,126^ Cattle slurry, for example, is enriched in degraded OM and OM resistant to degradation compared to farmyard manures.^125^ In addition, Kao et al. (2023) observed higher Se and S extractability after the application of urine compared to feces, particularly in low OM soil.^127^ In our data set, weak and nonsignificant positive correlations between manure input in the form of slurry and the proportion of NaOH-extractable organic Se and S in soil (Se, r_S_ = 0.30, P = 0.08; S, r_S_ = 0.30, P = 0.09) support the relevance of manure type and associated OM composition for Se and S speciation in soil. More directly, the strong connections between NaOH-extractable organic Se and S and PC1 of the OM composition data set indicate that specific SOM components, not simply overall SOC content, are key to their retention. The quality of SOM is thus more critical for retaining organic Se and S in soil than the total amount of SOC.

The strong influence of pH on the proportion of NaOH-extractable organic Se in F2+F3 in soil is partially related to its relationship with OM composition, reflected in the correlation between pH and PC1 separating fresh and/or poorly decomposed OM from degraded OM (r_S_ = −0.67, *P* < 0.0001, Figure 2a). In addition, pH is known to be strongly linked to SOM stabilization mechanisms,^91^ which can explain its influence on C and Se extractability by NaOH and the proportions of residual Se. Previous studies showed that OM tends to be more strongly stabilized at higher pH,^128^ and that NaOH extractability of C is specifically low in calcareous soils.^129,130^ Similarly, we observed negative correlations between pH and NaOH extractability of SOC as well as Se and S (Figure S6), resulting in a positive correlation between pH and residual Se and S (Se, r_S_ = 0.70, *P* < 0.0001; S, r_S_ = 0.90, *P* < 0.0001; Table S14; Figure 4). In calcareous soils, the immobilization of selenate added within incubation experiments was mainly due to the retention of Se on CaCO_3._ ^22^ This mechanism could explain the observed strong positive correlation between residual Se and Ca_Soil_, which originates either from carbonate-bearing parent material present in several Swiss regions or from liming.^50^ Similarly, coprecipitation of Se oxyanions can occur with Al/Fe/Mn-(hydr-)­oxides,^9^ which could be indicated in our data set by positive correlations between residual Se and Mn_Soil_ and Fe_Soil_. Furthermore, pH shapes microbial communities.^131^ In the incubation experiment of Vermeiren et al. (2025), the immobilized selenate recovered in the residual soil correlated positively with pH and further increased when microbial activity was stimulated by adding available C.^30^ This suggests that microbes were responsible for the increased stabilization of Se at higher pH, likely due to increased microbial production of Se(0).^132–135^ Overall, the higher proportion of residual Se in neutral to alkaline soils from our Swiss soil collection is potentially explained by stronger SOM stabilization, microbial Se(0) production, and increased Ca concentrations.

When considering each land use separately, retained Se (larger and aromatic organic Se (F2+F3) and residual Se) remained linked to PC1 of the OM data set and pH, but to variable degrees (Figure 4). The correlations were stronger in forest than in grassland and cropland soils, which was also seen for S (Figure S14). Forest soils span the largest gradients in OM composition and pH and are less frequently subjected to management interventions that introduce variability compared to agriculturally used sites. Many agricultural management practices are likely indirectly influencing Se mobility and plant availability by modifying soil pH and OM composition.^8^ Liming can enhance Ca_Soil_ and soil pH,^92^ which were both linked to retention of Se in soil in the form of residual Se, the least mobile Se fraction. In line with this, CaCO_3_ was found to negatively impact the capacity of crops to accumulate Se from soil.^136^ Tillage facilitates SOM decomposition,^92^ and thus likely also the decomposition of organic Se in F2+F3, depleting the reservoir of organic Se in soil but presumably resulting in more mobile and plant available Se­(IV) and small hydrophilic organic Se in F4. Application of organic amendments, such as plant residues and manure, can have dual effects on Se plant availability,^11,16–18^ which is potentially explainable by the OM composition of inputs and soil, as hypothesized earlier. Further research on how agricultural management practices influence Se speciation, including the different organic fractions detected by SEC-UV-ICP-MS/MS, could help to assess Se mobility and plant availability in agricultural soils. In particular, studies linking OM composition of inputs and soil with Se speciation in soil and soil solution and Se plant uptake are needed to disentangle the dual role of OM in enhancing or decreasing Se plant availability.^11,111^

**Figure 4:**
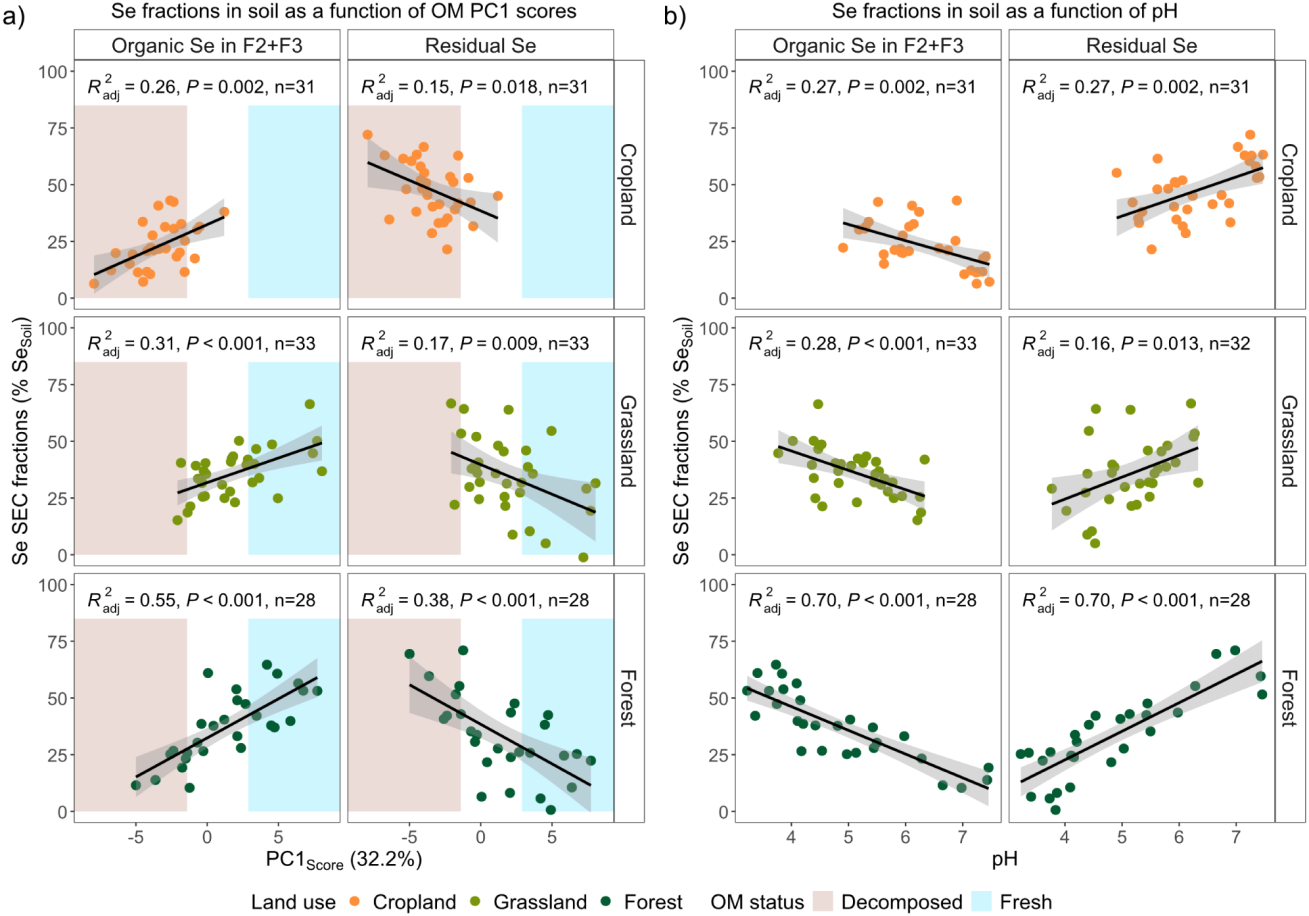
Larger and aromatic organic Se (F2+F3) and residual Se as proportions in soil as functions of a) OM PC1 scores derived from the PCA based on relative abundances of Py-groups, and b) pH per land use. The predicted lines from the linear model fit are shown in black and their 0.95 confidence intervals in gray. The adjusted coefficients of determination (R^2^_adj_), P-values (P), and number of samples (n) of the fitted linear models are reported. The background color in a) indicates the OM decomposition status along PC1 (decomposed versus fresh and/or poorly decomposed). The points represent the individual sites and are colored according to land use (cropland, grassland, forest).

We showed that the proportion of larger and aromatic organic Se (F2+F3) in soil that is retained but can become mobile and plant available upon decomposition is mainly linked to OM composition and pH, which could indicate different retention mechanisms. The influence of soil pH on Se extractability by NaOH implies that further optimization of the method to extract Se species is an important next step to pursue to extract organic Se compounds more comparably between soils, especially when calcareous and acidic soils are included. Soils could be pretreated with a decalcification step to obtain more comparable extracts.^137^ Additionally, Se speciation could be combined with the physical separation of OM into mineral-associated OM and particulate OM, as this separation helps to understand SOM stability and susceptibility to environmental changes.^49,82–84^ Furthermore, combining the SEC method with high-resolution molecular mass spectrometry remains open to unravel the molecular structure of present organic Se forms in soil.^13^

## Supplementary Material



## Data Availability

Data sets generated and analyzed during the current study are openly available in the Eawag Research Data Institutional Collection (https://doi.org/10.25678/000FFE).^138^

## Notes

Site information, extended methods and quality control results, additional visualizations of soil properties, NaOH extractability, SEC-UV-ICP-MS/MS data, Py-GC/MS data, and statistical outputs of correlation analyses (PDF)

## ⊥

Department of Physical Geography, Stockholm University, Svante Arrhenius väg 8, 106 91 Stockholm, Sweden

## #

METAS, Swiss Federal Institute of Metrology, Laboratory Organic Analysis and References, Lindenweg 50, 3003 Bern-Wabern, Switzerland
