# Supplementary material for: Organic Selenium Quality and Quantity in Soils Are Related to Organic Matter Composition and Driven by Land Use
Source: Environ Sci Technol. 2026 Mar 4;60(10):7897–911. doi: 10.1021/acs.est.5c15430 (PMC13001097; doi:10.1021/acs.est.5c15430)
Supplement: Supplementary file 1 [file es5c15430_si_001.pdf]

Organic selenium quality and quantity in soils are related to organic matter composition and driven by land use

Maja B. Siegenthaler[a,b,c,#,*], Lenny H. E. Winkel[a,b,*], Reto G. Meuli[c], Julie Tolu[a,b,$,*]

[a] Eawag, Swiss Federal Institute of Aquatic Science and Technology, Department of Water Resources and Drinking Water (W+T), Überlandstrasse 133, 8600 Dübendorf, Switzerland

[b] ETH Zurich, Swiss Federal Institute of Technology, Department of Environmental Systems Sciences (D-USYS), Institute of Biogeochemistry and Pollutant Dynamics (IBP), Group of Inorganic Environmental Geochemistry, Universitätstrasse 16, 8092 Zurich, Switzerland

[c] Agroscope, Agroecology and Environment, Soil Quality and Soil Use, Reckenholzstrasse 191, 8046 Zurich, Switzerland

Present addresses:

[#] M.B.S.: Department of Physical Geography, Stockholm University, Svante Arrhenius väg 8, 106 91Stockholm, Sweden

[$] J.T.: METAS, Swiss Federal Institute of Metrology, Laboratory Organic Analysis and References, Lindenweg 50, 3003 Bern-Wabern, Switzerland

* Corresponding authors:

* Email: maja.siegenthaler@natgeo.su.se

* Email: lenny.winkel@eawag.ch

* Email: julie.tolu@metas.ch

Summary: 39 pages, 14 figures, 14 tables, 7 supplementary methods

Table of Contents

Supplementary Materials and Methods S3
Figure S1. Site location, elevation, temperature, precipitation S3
Table S1. Soil types S3
Method S1. Soil sampling and pre-treatments S4
Table S2. Data origin of soil properties S5
Method S2. Microwave-assisted acid digestion of soils S6
Method S3. Total element quantification in acid digests and NaOH extracts by ICP-MS/MS S6
Method S4. NaOH extraction of soils and Se and S speciation in NaOH extracts S6
Table S3. ICP-MS/MS analysis acquisition information S8
Table S4. Aqueous certified reference materials S9
Table S5. Soil certified reference materials in acid digests S10
Table S6. One soil from the Swiss soil collection as quality control S11
Table S7. Hawaii soils to validate NaOH extraction S12
Method S5, Table S8, Figure S2. On-line isotope dilution calculation including interferences and mass bias corrections S13
Figure S3. SEC chromatogram S15
Method S6. Organic matter molecular composition by Py-GC/MS S16
Table S9. Organic matter compounds identified by Py-GC/MS S17
Figure S4. Py-GC/MS identified peak area compared to SOC and TN S21
Table S10. Analytical reproducibility of Py-GC/MS results per biochemical class S21
Method S7. Statistical analysis and data visualization S22
Supplementary Results S23
Figure S5. SOC, TN, C/N, pH, Se_{Soil} and S_{Soil} per land use S23
Table S11. Correlations between soil Se and S concentrations and soil properties S24
Figure S6. NaOH extractabilities of Se, S, and soil organic carbon (SOC) S25
Figure S7. Comparison of free Se(IV) concentrations determined by SEC-UV-ICP-MS/MS and AEC-ICP-MS/MS S25
Figure S8. Se and S species concentrations in soil per land use S26
Table S12. Decomposition status assigned to organic matter Py-groups S27
Figure S9. Py-groups per land use S29
Figure S10. Se species as proportions in NaOH extracts as functions of OM PC1 per land use S30
Table S13. Correlations between Se and S species as proportions in NaOH extracts and OM PCs and soil properties S31
Figure S11. Se and S species as proportions in NaOH extracts as functions of pH S32
Figue S12: Residual Se and S S33
Table S14. Correlations between Se and S species as proportions in soil and OM PCs and soil properties S34
Figure S13. Organic Se proportions in soil as a function of SOC for the Swiss soil collection and the Hawaii soils studied by Tolu et al. (2022) S35
Figure S14. S F2+F3 and residual S as proportions in soil as functions of organic matter PC1 and pH per land use S36
References S37

Supplementary Materials and Methods

Figure S1. Site location, elevation, temperature, precipitation

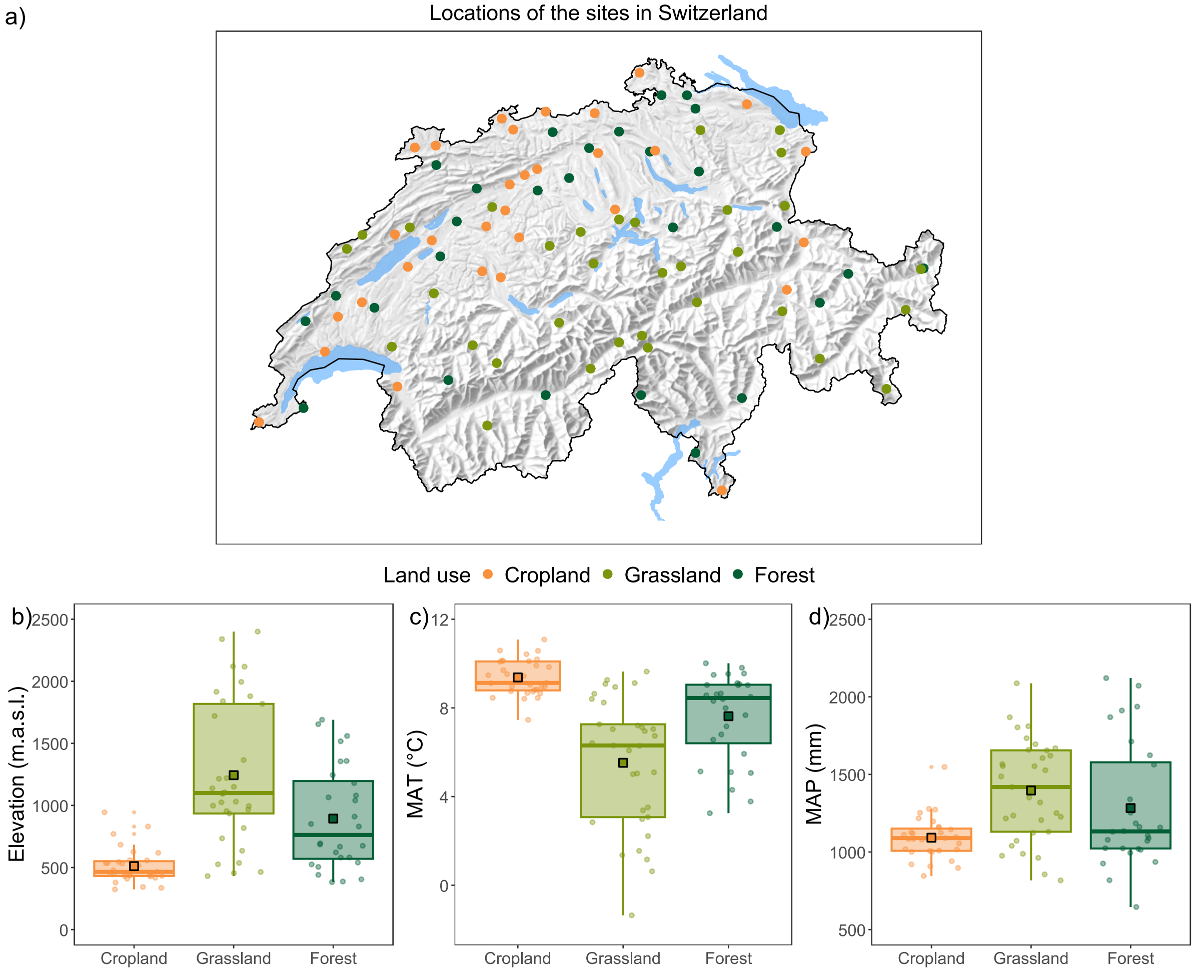

Figure S1. a) locations of the sites in Switzerland, and their b) elevation (meters above sea level, m.a.s.l.), c) mean annual temperature (MAT, °C), and d) mean annual precipitation (MAP, mm) per land use. Sites are colored according to land use (cropland, grassland, forest). MAP and MAT are based on meteorological data for 1981-2010 from the Federal Office of Meteorology and Climatology MeteoSwiss. The Swiss relief is based on DHM 25 from the Federal Office of Topography swisstopo.

Table S1. Soil types

Table S1. Soil types according to the Swiss soil classification and the corresponding number of sites included in this study.

Soil type (Swiss soil classification)	Number of sites	Soil type (Swiss soil classification)	Number of sites
Braunerde	24	Braunerde-Pseudogley	3
Saure Braunerde	18	Buntgley	3
Parabraunerde	6	Phaeozem	3
Rendzina	6	Pseudogley	2
Fluvisol	5	Aue	1
Kalkbraunerde	5	Fahlgley	1
Podsol	5	Halbmoor	1
Braunerde-Gley	4	Moor	1
Braunpodsol	3	Regosol	1

Method S1. Soil sampling and pre-treatments

In each sampling campaign of the Swiss Soil Monitoring Network, four topsoil replicates, each consisting of a composite of 25 subsamples, were collected in an area of 10x10 m^2 using a gouge auger (Eijkelkamp, 2.5 cm diameter) by applying a stratified random sampling design.[1,2] The analyses conducted in this study were performed on one archived topsoil replicate sampled between 2010-2017.

Bulk soil properties, determined using Swiss standard methods,[3] were taken from the Swiss Soil Monitoring Network database (Table S2). Bulk soil properties measured on the exact same topsoil replicate were used in this study when available (i.e., for pH, soil organic carbon (SOC), total nitrogen (TN), and for the majority of soil texture data; Table S2). Otherwise, data for the topsoil collected at the same site at the respective first sampling campaign, taking place between 1985-1989 for 82 sites and 1995-1999 for three sites, was integrated (i.e., for potential cation exchange capacity (CEC_{pot}), amorphous Fe oxides (Fe_{oxa}), minority of soil texture data; Table S2). For seven sites, the selected sampling campaign was the respective first sampling campaign and thus no additional data was available (i.e., no data for CEC_{pot}, Fe_{oxa}). Bulk soil properties were determined on samples dried at 40°C and sieved at 2 mm. Soil pH was determined in 0.01 M $CaCl_2$ after 2h of extraction (10 g soil in 25 mL). Total carbon (TC) and nitrogen (TN) were determined by dry combustion (Truscpec CN, Leco). Inorganic carbon (IC) was obtained by HCl digestion and volumetric metering of the produced CO_2. Soil organic carbon (SOC) was determined by subtracting IC from TC. Soil texture (clay and silt content) was determined by fine earth sedimentation after removing the humus fraction with H_2O_2. Potential cation exchange capacity (CEC_{pot}) was determined by atomic absorption spectrometry in 0.1 M $BaCl_2$ and 0.03 M triethanolamine after extraction at 45°C for 12-18 hours. Amorphous Fe oxides (Fe_{oxa}) were determined by atomic absorption spectrometry after extraction with an oxalate solution (pH 3).

Basal soil respiration data for the topsoil collected at the same site at the latest sampling campaign, taking place between 2018-2023, was also included (Table S2). This analysis was performed on three topsoil replicates, each consisting again of 25 subsamples collected in an area of 10x10 m^2 following the same sampling design.[4] For this analysis, the soils were kept fresh, sieved at 2 mm, and pre-incubated to reach an equilibrium state with standardized water content . Basal soil respiration was then quantified on equilibrated soils by trapping the CO_2 released between 24-29 hours of incubation at 25 °C in NaOH and by back-titrating with HCl. Since basal soil respiration was measured under optimal conditions, it is likely not reflecting in situ microbial activity.

In addition, volumetric soil samples were taken at each edge of the area using an HS impact probe (GreenGround, 4.8 cm diameter, 25 cm length, sampling 0-20 cm depth) to determine the apparent density of the fine earth (ADFE, mass of fine earth, i.e., particles <2 mm divided by sample volume).[5] In this study, the mean values of the additional volumetric soil samples from the same sampling campaign were integrated (Table S2).

Table S2. Data origin of soil properties

Table S2. Data origin of soil properties from the Swiss Soil Monitoring Network and the Geochemical Soil Atlas of Switzerland integrated in this study.[6] The topsoil replicates used in this study (same replicate) were sampled within 2010-2017. The topsoil replicates from the respective first sampling campaign (first campaign) were sampled within 1985-1989 for 82 sites and 1995-1999 for three sites. The topsoil replicates from the latest sampling campaign (latest campaign) were sampled between 2018-2023. Soil properties are soil organic C (SOC), total nitrogen (TN), soil texture, pH, apparent density of the fine earth (ADFE), potential cation exchange capacity (CEC_{pot}), amorphous Fe oxides (Fe_{oxa}), concentrations of Ca and Mg in soil (Ca_{Soil} and Mg_{Soil}).

	**Data origin**					
Sampling campaign	Same	Same	First	First	Latest	No data
Sample	Same replicate	Mean of vol. samples	Mean of 4 replicates	1 replicate	Mean of 3 replicates	
	**Soil properties**					
SOC	92					
TN	92					
Soil texture	78		6	6		2
pH	92					
ADFE		92				
CEC_{pot}			67	18		7
Fe_{oxa}			64	18		10
Ca_{Soil}	87					5
Mg_{Soil}	87					5
Basal respiration					90	2

Method S2. Microwave-assisted acid digestion of soils

Fifty mg of dried and finely ground soil was digested in duplicates with 2 ml HNO_3 (65%, Suprapur, Roth), 1 ml H_2O_2 (30%, for trace analysis, Supelco) and 0.3 ml HF (40%, Suprapur, Merck) at 240°C and 140 bar for 20 min in a microwave (UltraCLAVE 4, MLS GmbH). The Teflon vials used for the digestion were previously acid cleaned, first in an acid bath (1% HNO_3) and then in a cleaning digestion run (4 ml H_2O and 4 ml HNO_3 (65%, Suprapur, Roth). All digests were made up to 50 ml with ultrapure water (resulting in 1:1000 soil:solution ratio) and centrifuged (3118 x g for 15 min, Rotina 380, Hettich Zentrifugen) before ICP-MS/MS analysis.

Method S3. Total element quantification in acid digests and NaOH extracts by ICP-MS/MS

Concentrations of Se, S, Fe, and Mn in acid digests and NaOH extracts (Method S4, diluted 25-100 times) were quantified in analytical triplicates using an 8900 ICP-MS/MS (Agilent Technologies) equipped with a prepFAST autosampler/autodiluter (Elemental Scientific Inc.), a Micromist nebulizer, a double-pass Scott chamber cooled to 2°C, a 2.5 mm ID torch, and Ni skimmer and sampler cones. All ICP-MS/MS parameters were optimized prior to analysis using a tuning solution containing 10 µg L^{-1} of Li, Y, Co, Ce, and Te. Signal stability during analysis was monitored using an internal standard solution containing 1% HNO_3, 1 mg L^{-1} Sc, 100 µg L^{-1} Rh, and 100 µg L^{-1} Lu. The acquisition parameters used for each element are given in Table S3. In the prepFAST, 4% HNO_3 was used as carrier and diluent for the analysis of the digests and ultrapure water for the analysis of NaOH extracts. Elements were quantified by external standard curves (Se, S, Fe single standards, J.T. Baker and Bernd Kraft; multielement standard solution V for ICP, Sigma-Aldrich) prepared in 4% HNO_3 for the acid digests and in 0.1 M NaOH for the NaOH extracts. Results obtained for the QCs are reported in Tables S4-S7.

Concentrations of Ca and Mg (Ca_{Soil} and Mg_{Soil}) for the exact same topsoil replicate were taken from the Geochemical Soil Atlas of Switzerland (Table S2). They were determined by digestion with a modified aqua regia solution (HNO_3:HCl:H_2O 1:1:1) and ICP-MS analysis.[6]

Method S4. NaOH extraction of soils and Se and S speciation in NaOH extracts

Dried and finely ground soil was directly extracted with 0.1 M NaOH with a 1:33 soil:solution ratio by overhead shaking at 250 rpm for 24 hours at room temperature, as optimized by Tolu et al. (2011).[7] All extracts were centrifuged (3118 x g for 30 min, Rotina 380, Hettich Zentrifugen), filtered at 0.45 µm (Perfect-Flow®, Nylon membrane, BGB), and stored at 4°C for i) max. two weeks for Se speciation (Se(IV) and Se(VI) quantification) by anion exchange chromatography (AEC) coupled to ICP-MS/MS; ii) max. three days for Se and S speciation by size exclusion chromatography (SEC) coupled to UV and ICP-MS/MS; iii) max. one week for dissolved organic carbon (DOC) quantification by a TOC analyzer (TOC-L CSH, Shimadzu); and iv) max. one month for total element quantification by ICP-MS/MS.

Selenite (SeO_3^{2-}(IV)) and selenate SeO_4^{2-}(VI) were quantified in ten times diluted NaOH extracts by anion exchange chromatography (AEC) coupled to ICP-MS/MS using an Agilent 1260 Infinity II high-performance liquid chromatography (HPLC) system and the ICP-MS/MS described before. A PRP-100X column (5 µm, 150x4.1 mm, Hamilton Company Inc.) with an in-line filter was used with a mobile phase containing 20 mM ammonium citrate (dibasic 98% and tribasic ≥97% (titration), Sigma-Aldrich) and 2% MeOH (Optima™ LC/MS grade, Thermo Scientific™) at pH 5.2 (fixed with 25% ammonia, Emsure®, Merck).[7,8] The flow rate was of 1.25 mL min^{-1} and injection volume was of 100 µl. ICP-MS/MS was operated as described before, and Se was detected in MS/MS mode with 5.5 mL min^{-1} H_2 in the C/RC, with *m/z* 78->78 and 0.1 s acquisition time. Se species were quantified by external standard curves (SeO_3^{2-}(IV) 99.999% in 2% HNO_3 and SeO_4^{2-}(VI) 99.99% in 0.1% HNO_3, Spectracter) prepared in the corresponding NaOH matrix, and each soil extract was analyzed in duplicate. A solution containing 1 mg L^{-1} Sc, 100 µg L^{-1} Rh, and 100 µg L^{-1} Lu was added post-column using a T-piece and the ICP-MS/MS peristaltic pump to check for signal stability during the runs. Results obtained for the QCs are reported in Tables S6 and S7.

NaOH extracts were analyzed undiluted by SEC-UV-ICP-MS/MS using an Agilent 1260 Infinity II HPLC system equipped with an Agilent diode array detector (DAD) and the ICP-MS/MS described above. The operating conditions were as described in Tolu et al. (2022).[8] Briefly, a series of OH-pak SB-803 HQ and OH-pak SB-802.5 HQ SEC columns (Shodex) was used with the guard column (OH-pak SB-G 6B, Shodex). The mobile phase was 5 mM ammonium nitrate (Emsure®, Merck) at pH 9 (fixed with 25% ammonia, Emsure®, Merck), delivered at a flow rate of 1 mL min^{-1}, and injection volume was of 100 µl. Each NaOH extract was measured twice with UV detection at 254 nm but with 2 different ICP-MS/MS operating conditions: i) with 5.5 mL min^{-1} H_2 in the C/RC to measure Se together with As, Cu, Fe; and ii) with 1 mL min^{-1} H_2 and 30% O_2 in the C/RC to measure S together with Se (Table S3). A solution containing 118 ppb $^{78}SeO_3^{2-}$(IV) (prepared from Isoflex ^{78}Se(0) standard), 100 µg L^{-1} Sc and 100 µg L^{-1} Y was added post-UV detector using a T-piece and the ICP-MS/MS peristaltic pump to quantify Se in SEC-peaks by on-line isotope dilution and check for signal stability during the runs (Method S5). The peak deconvolution of Se mass flow chromatograms and S intensity chromatograms was conducted with the peak analyzer function (Fit peaks pro) of OriginPro 2023 software (OriginLab Corporation) following the procedure of Laborda et al. (2011).[9] In brief, we first set the baseline of the chromatogram, then manually indicated the position of the individual peaks, and finally the individual peaks were fitted step-wise to the overall chromatogram. The deconvoluted Se and S SEC peaks were combined into fractions that were defined by the elution time, the co-elution with other elements and the UV signal as described previously.[8] The fractions were as follows: F1 (organo)mineral nanoparticles; F2 larger, more negatively charged, Fe-enriched organic matter; F3 smaller, less negatively charged, aromatic organic matter; F4 small hydrophilic organic matter; F5 free oxyanions. The sum of fractions F2, F3, and F4 is representing all organic Se or S compounds extracted by NaOH. Results obtained for the QCs are reported in Tables S6 and S7.

Proportions of Se and S SEC fractions in NaOH extracts (% Se_{NaOH} and % S_{NaOH}) were calculated by dividing the mass of Se, respectively the chromatogram area of S, of the respective fraction by the total mass or chromatogram area of the sample. Proportions of Se and S SEC fractions in soil (% Se_{Soil} and % S_{Soil}) were calculated by multiplying the proportions in NaOH extracts with the extractability by NaOH. Concentrations of SEC fractions in soil (µg Se kg^{-1} and mg S kg^{-1}) were calculated based on 100% recovery of total Se and S extracted by NaOH in SEC and multiplying the proportion of the SEC fractions in NaOH with the total element concentrations in NaOH extracts. Residual Se and S concentrations (µg Se kg^{-1} and mg S kg^{-1}) were calculated by subtracting the concentrations of total Se and S extracted by NaOH from the total Se and S in soil. Residual Se and S proportions in soil (% Se_{Soil} and % S_{Soil}) were calculated by dividing the residual Se and S by the total Se and S in soil.

Table S3. ICP-MS/MS analysis acquisition information

Table S3. Acquisition parameters for each element measured in ICP-MS/MS analysis of elements in soil digests and NaOH extracts and SEC-UV-ICP-MS/MS of NaOH extracts.

Element	*m/z*	Scan Type	Gas	Gas flow rate	Integration time<br>s
**ICP-MS/MS analysis of elements in soil digests and NaOH extracts**					
Fe	56	single-quad	He	5.5 mL He min^{-1}	0.0201 (for 1 of 4 batches)
Fe	56	single-quad	He	5.5 mL He min^{-1}	0.0999 (for 3 of 4 batches)
Mn	55 → 55	MS/MS	H_2	5.0 mL H_2 min^{-1}	0.0999
S	32 → 48	MS/MS	O_2	1.0 ml H_2 min^{-1} 30% O_2	0.0201 (for 1 of 4 batches)
S	32 → 48	MS/MS	O_2	1.0 ml H_2 min^{-1} 30% O_2	0.0501 (for 3 of 4 batches)
Se	80 → 96	MS/MS	O_2	1.0 ml H_2 min^{-1} 30% O_2	0.9999 (for 2 of 4 batches)
Se	80 → 96	MS/MS	O_2	1.0 ml H_2 min^{-1} 30% O_2	0.2001 (for 1 of 4 batches)
Se	80 → 96	MS/MS	O_2	1.0 ml H_2 min^{-1} 30% O_2	2.0001 (for 1 of 4 batches)
**SEC-UV-ICP-MS/MS of NaOH extracts**					
As	75 → 75	MS/MS	H_2	5.5 mL H_2 min^{-1}	0.1000
Cu	63 → 63	MS/MS	H_2	5.5 mL H_2 min^{-1}	0.0500
Fe	56 → 56	MS/MS	H_2	5.5 mL H_2 min^{-1}	0.0100
S	32 → 48	MS/MS	O_2	1.0 ml H_2 min^{-1} 30% O_2	0.0500
Se	77 → 77	MS/MS	O_2	1.0 ml H_2 min^{-1} 30% O_2	0.1000
Se	78 → 78	MS/MS	O_2	1.0 ml H_2 min^{-1} 30% O_2	0.1000
Se	80 → 80	MS/MS	O_2	1.0 ml H_2 min^{-1} 30% O_2	0.1000

Table S4. Aqueous certified reference materials

Table S4. Certified and measured element concentrations as well as resulting element recoveries (mean, M and standard deviation, SD) for the two aqueous certified reference materials (NIST 1643f Trace elements in water and Multielement Anion HPCE Standard Solution 29235) used to validate the ICP-MS/MS analysis of elements in acid digests and NaOH extracts. These two CRMs were diluted in the corresponding digest or NaOH extract matrix (HNO_3 and NaOH) in replicates (n=9 for the analysis of acid digests and n=4 for the analysis of NaOH extracts). Both acid digests and NaOH extracts were analyzed in two batches.

			Batch 1					Batch 2				
**Element**	**Certified element conc.**		**Measured element conc.**		**Recovery**		**Error**	**Measured element conc.**		**Recovery**		**Error**
	µg L^{-1}		µg L^{-1}		**%**		%	µg L^{-1}		**%**		%
	**M**	SD	**M**	SD	**M**	SD	M	**M**	SD	**M**	SD	M
**NIST 1643f Trace elements in water – diluted in HNO_3 (n=9)**												
**Mn**	**37.1**	0.6	**37**	5	**100**	15	0	**40**	2.	**108**	7	8
**Fe**	**93.4**	0.8	**80**	57	**85**	61	-15	**107**	50	**115**	54	15
**Se**	**11.7**	0.1	**11.0**	0.5	**94**	5	-6	**11.4**	0.7	**98**	6	-2
**Multielement Anion HPCE Standard Solution 29235 – diluted in HNO_3 (n=9)**												
**S**	**32593**	1630	**32779**	3313	**101**	11	1	**32700**	2486	**100**	9	0
**NIST 1643f Trace elements in water – diluted in NaOH (n=4)**												
**Mn**	**37.1**	0.6	**36.1**	0.7	**97**	2	-3	**36**	5	**96**	13	-4
**Fe**	**93.4**	0.8	**90**	11	**96**	12	-4	**110**	98	**117**	104	17
**Se**	**11.7**	0.1	**9.8**	0.9	**84**	8	-16	**10**	1	**83**	9	-17
**Multielement Anion HPCE Standard Solution 29235 – diluted in NaOH (n=4)**												
**S**	**32593**	1630	**26758**	320	**82**	4	-18	**29138**	1282	**89**	6	-11

Table S5. Soil certified reference materials in acid digests

Table S5. Certified and measured element concentrations as well as resulting element recoveries (mean, M and standard deviation, SD) for the four soils certified reference materials (n=6 for Sigma-Aldrich CRM-044 Silt Loam 1, NIST SRM-2709a San Joaquin soil, CNMR GBW 07405 Chinese Yellow-red soil, and n=4 for CNRM GBW 07408 Chinese Loess) used to validate the digestion procedure and subsequent quantification of elements in acid digests by ICP-MS/MS.

**Element**	**Certified element conc.** mg kg^{-1}		**Measured element conc.** mg kg^{-1}		**Recovery** %		**Error** %
	**M**	SD	**M**	SD	**M**	SD	M
**Sigma-Aldrich CRM-044 Silt Loam 1**							
**Mn**	**204**	14	**256**	15	**126**	11	26
**Fe**	**3180**	284	**4010**	310	**126**	15	26
**Se**	**81**	7	**76**	7	**93**	11	-7
**NIST SRM-2709a San Joaquin soil**							
**Mn**	**529**	18	**575**	21	**109**	5	9
**Fe**	**33630**	700	**32581**	1316	**97**	4	-3
**Se**	**1.5**	NA	**1.4**	0.1	**94**	6	-6
**CNMR GBW 07405 Chinese Yellow-red soil**							
**S**	**410**	54	**438**	17	**107**	15	7
**Mn**	**1360**	71	**1445**	47	**106**	7	6
**Fe**	**88267**	1259	**78919**	4306	**89**	5	-11
**Se**	**1.6**	0.2	**1.8**	0.1	**112**	17	12
**CNRM GBW 07408 Chinese Loess**							
**S**	**126**	NA	**141**	13	**112**	NA	12
**Mn**	**650**	23	**709**	39	**109**	7	9
**Fe**	**31334**	350	**30458**	1166	**97**	4	-3
**Se**	**0.10**	0.01	**0.05**	0.03	**55**	30	-45

Table S6. One soil from the Swiss soil collection as quality control

Table S6. Measured Se and S concentrations (mean, M and standard deviation, SD) as well as the resulting relative standard deviation (RSD) obtained for one randomly selected soil from the Swiss soil collection that was analyzed in 5-6 replicates. These data allow to estimate the variability of total soil Se concentrations (determined by ICP-MS/MS after acid digestion), total Se concentrations in NaOH soil extracts (determined by ICP-MS/MS), Se(IV) concentrations in NaOH extracts (determined by AEC-ICP-MS/MS), and Se proportions in SEC fractions in NaOH soil extracts (determined by SEC-UV-ICP-MS/MS with on-line isotopic dilution and peak deconvolution). The calculation of the standard deviation (SD) and subsequent relative standard deviation (RSD) includes error propagation of the standard deviations of analytical and methodological replicates.

Parameter	Unit (M and SD)	Method	Number of Replicates	M	SD	RSD (%)
**Se Soil**	µg kg^{-1}	Acid digestion + ICP-MS/MS	5	**366**	36	**10**
**Se NaOH**	µg kg^{-1}	NaOH extraction + ICP-MS/MS	6	**170**	16	**10**
**Se NaOH**	% Se Soil		6	**47**	6	**14**
**Se(IV)**	µg kg^{-1}	NaOH extraction + AEC-ICP-MS/MS	6	**50**	8	**16**
**Se(IV)**	% Se NaOH		6	**29**	6	**20**
**Se F5: Se oxyanions**	% Se NaOH	NaOH extraction + SEC-UV-ICP-MS/MS	6	**31**	3	**11**
**Se sum organic fractions**			6	**69**	3	**5**
**Se F2**			6	**13**	5	**39**
**Se F3**			6	**27**	5	**17**
**Se F4**			6	**29**	3	**12**
**S Soil**	mg kg^{-1}	Acid digestion + ICP-MS/MS	5	**793**	63	**8**
**S NaOH**	mg kg^{-1}	NaOH extraction + ICP-MS/MS	6	**193**	17	**9**
**S NaOH**	% S Soil		6	**24**	3	**12**
**S F5: S oxyanions**	% S Soil	NaOH extraction + SEC-UV-ICP-MS/MS	6	**26**	1	**3**
**S sum organic fractions**			6	**74**	1	**1**
**S F2**			6	**33**	7	**20**
**S F3**			6	**35**	5	**15**
**S F4**			6	**6**	2	**38**

Table S7. Hawaii soils to validate NaOH extraction

Table S7. Measured Se and S concentrations (mean, M and standard deviation, SD) in two Hawaii soils (n=3) previously studied by Tolu et al. (2022)[8] to validate the NaOH extraction and subsequent quantification of elements in soil extracts by ICP-MS/MS as well as subsequent Se and/or S speciation analyses by AEC-ICP-MS/MS and SEC-UV-ICP-MS/MS.

Element	Tolu et al. (2022) Measured conc. (n=1)		Measured conc. (n=3)		Recovery		Error
	**M**	SD	**M**	SD	**M**	SD	M
**Hawaii soil D1S1S2**							
	µg kg^{-1}		%		%		%
**Se NaOH**	**1025**	5	**957**	98	**93**	10	-7
**Se(IV) AEC**	**644**	51	**577**	24	**90**	8	-10
	%		%		%		%
**Se(IV) AEC**	**63**	5	**61**	7	**97**	13	-3
**Se F5 SEC: Se oxyanions**	**56.1**	0.7	**67**	1	**119**	3	19
**Organic Se SEC**	**44**	1	**33**	1	**76**	3	-24
	mg kg^{-1}		%		%		%
**S NaOH**	**707**	24	**530**	61	**75**	9	-25
	%		%		%		%
**S F5 SEC: S oxyanions**	**28.1**	0.1	**27.0**	0.5	**96**	2	-4
**Organic S SEC**	**71.7**	0.3	**72.9**	0.7	**102**	1	2
**Hawaii soil D3S1S2**							
	µg kg^{-1}		%		%		%
**Se NaOH**	**2529**	133	**2302**	251	**91**	11	-9
**Se(IV) AEC**	**438**	3	**447**	28	**102**	6	2
	%		%		%		%
**Se(IV) AEC**	**17.3**	0.9	**20**	2	**113**	14	13
**Se F5 SEC: Se oxyanions**	**14.6**	0.2	**18**	1	**124**	10	24
**Organic Se SEC**	**79.9**	0.9	**82**	1	**103**	2	3
**Se F1 SEC**	**3.2**	0.6	**No peak**				
	mg kg^{-1}		%		%		%
**S NaOH**	**864**	26	**659**	66	**76**	8	-24
	%		%		%		%
**S F5 SEC: S oxyanions**	**6.38**	0.05	**8**	4	**133**	59	33
**Organic S SEC**	**91.0**	0.3	**92**	4	**101**	4	1

Method S5, Table S8, Figure S2. On-line isotope dilution calculation including interferences and mass bias corrections

From the post UV-detector addition of $^{78}Se(IV)$ and the acquisition of Se and bromine (Br) isotopes by ICP-MS/MS during the SEC-UV-ICP-MS/MS analysis of all NaOH extracts, the Se mass flow chromatogram (M_s), which enables to quantify Se in each SEC peak, was determined following the steps and equations described below and in Tolu et al. (2022).[8]

Step 1. Interferences corrections

For each data point along the SEC elution, the intensities (I) of ^{78}Se and ^{80}Se were corrected for $^{77}Se^{1}H^{+}$ and $^{79}Br^{1}H^{+}$ interferences that are formed in the plasma using the following equations:

$^{77}Ic = ^{77}I – (fSe * ^{76}I)$
$^{78}Ic = ^{78}I – (fSe * ^{77}Ic)$
$^{79}Ic = ^{79}I – (fSe * ^{78}Ic)$
$^{80}Ic = ^{80}I – (fBr * ^{79}Ic)$

^{x}I is the gross signal intensity measured at the m/z x (x=77, 78, 79, or 80) with 77, 78, 80 being Se isotopes and 79 being a Br isotope.
^{x}Ic (x=77, 78, 79, or 80) is the signal intensity of the Se or Br isotope after correction for SeH^{+} or BrH^{+} interferences.
fSe is the factor of SeH^{+} formation determined by measuring the m/z ratio 83/82 ($^{82}Se^{1}H^{+}/^{82}Se$) in a 20 µg L^{-1} Se standard of natural abundance by ICP-MS/MS (Table S8).
fBr is the factor of BrH^{+} formation determined by measuring the m/z ratio 80/79 ($^{81}Br^{1}H^{+}/^{81}Br$) ratios in a 50 µg L^{-1} Br standard of natural abundance (Table S8).

The values of fSe, fBr, and mass bias K used to calculate the Se mass flow chromatograms were obtained on the same ICP-MS/MS between the two SEC-UV-ICP-MS/MS measurement runs. fSe, fBr and K were determined in a 20 µg L^{-1} Se standard of natural abundance and a 50 µg L^{-1} Br standard of natural abundance by measuring nine replicates in analytical triplicates.

Table S8. Selenium hydridation (fSe, %) and bromine hydridation (fBr, %) factors, as well as mass bias (K, %) used for the on-line isotopic dilution calculation. Mean (M) standard deviation (SD) and relative standard deviation (RSD, %) including error propagation of analytical triplicates and replicate (n=9) variation.

	**fSe** %			**fBr** %			**K** %	
M	SD	RSD	M	SD	RSD	M	SD	RSD
0.10	0.03	27.5	0.44	0.03	6.6	2.5	0.1	4.4

Step 2. Mass bias corrections

For each data point along the SEC elution, the ratio $^{80}Se/^{78}Se$ ($R_{sample, measured}$) was calculated using the corrected intensities of ^{78}Se and ^{80}Se, and this ratio was corrected for mass bias using the following equation.

$R_{sample, corrected}= R_{sample, measured} * e^{\wedge}(- K * \Delta M_{80/78})$

$R_{sample, measured}$ is the $^{80}Se/^{78}Se$ ratio calculated after correcting ^{78}Se and ^{80}Se intensities for interferences.
K is the mass bias factor determined from the analyses of a 20 µg L^{-1} natural abundance Se standard.
$\Delta M_{80/78}$ is the mass difference between ^{80}Se and ^{78}Se ($\Delta M_{80/78} = 2$).

More precisely, to determine K, the Napierian logarithm of the relative error of the experimentally measured isotopic ratio $^{78}Se/^{80}Se$ in the natural abundance Se standard ($R_{experimental}$) divided by the theoretical natural abundance ratio ($R_{theoretical}$) is plot against the mass difference (ΔM) between the isotope of reference (here ^{80}Se) and the other monitored isotopes (Figure S2). The result is a linear relationship and the mass bias factor (K) derived from the slope of the regression line. For $R_{theoretical}$ the values reported by CIAAW in 2021 (Isotopic compositions of the elements 2021, available online at www.ciaaw.org.) were used.

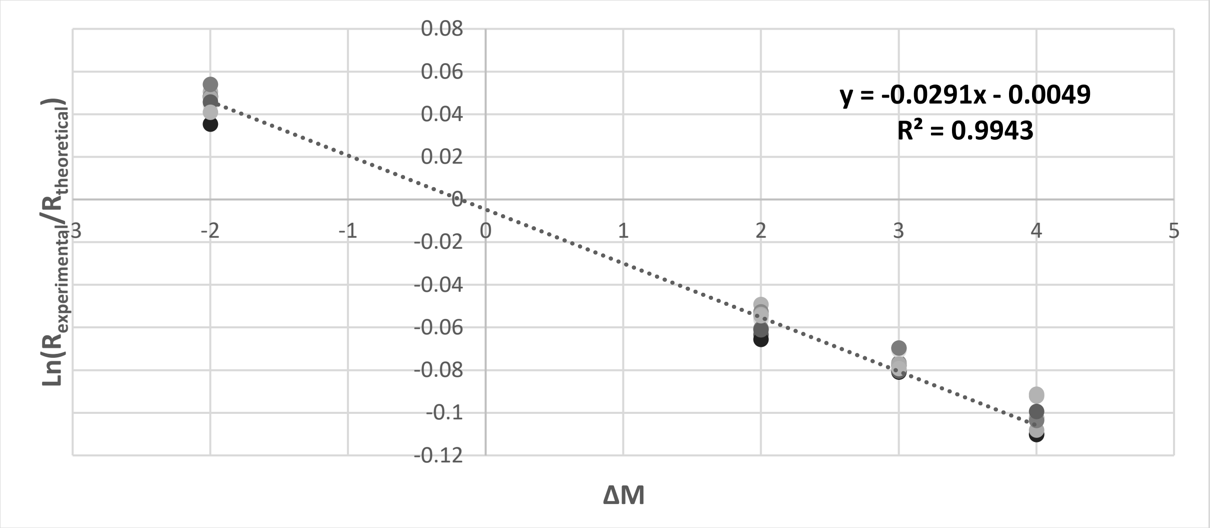

Figure S2. Determination of K from the linear relationship between $Ln(R_{experimental}/R_{theoretical})$ and the mass difference (ΔM) between the monitored isotopes (i.e., ^{76}Se, ^{77}Se, ^{78}Se and ^{82}Se) and the ^{80}Se reference isotope, from the analyses of a 20 µg L^{-1} natural abundance Se standards.

Step 3. Isotope dilution calculation to create the mass flow chromatogram

The Se mass flow chromatogram is created by calculating M_s (ng min^{-1}) for each data point along the SEC elution using the following equation:

$$M_s = C_{sp} * d_{sp} * f_{sp} * (AW_s/AW_{sp}) * (A^{78}{}_{sp}/A^{80}{}_{s}) * (R_m - R_{sp})/(1-R_m*R_s)$$

C_{sp} is the concentration of $^{78}Se(IV)$ in the spike solution added post-UV detector (ng g^{-1}), i.e., of 117.84 ng g^{-1} in this study.
d_{sp} is the density of the $^{78}Se(IV)$ spike solution (g mL^{-1}), i.e., of 1 g mL^{-1}.
f_{sp} is the flow rate to which the $^{78}Se(IV)$ spike solution is added post-UV detector (mL min^{-1}), i.e., of 0.068 mL min^{-1} in this study.
AW_s is the atomic weight of selenium in the sample, i.e., 78.971 (CIAAW, 2021).
AW_{sp} is the atomic weight of selenium in the $^{78}Se(IV)$ spike solution, i.e., 77.92 (CIAAW, 2021) in this study.
$A^{78}{}_{sp}$ is the abundance of ^{78}Se in the added $^{78}Se(IV)$ spike solution, i.e., of 99.50 in this study.
$A^{80}{}_{s}$ is the abundance of ^{80}Se in the sample, i.e., natural abundance which is of 49.80% (CIAAW, 2021).
R_m is the $^{80}Se/^{78}Se$ ratio after interferences and mass bias corrections.
R_{sp} is the $^{80}Se/^{78}Se$ ratio in the added $^{78}Se(IV)$ spike solution, i.e., of 0.0033 in this study.
R_s is the $^{78}Se/^{80}Se$ ratio in the sample, i.e., of 0.4757 (CIAAW, 2021).

Figure S3. SEC chromatogram

Figure S3. Element and ultraviolet (UV) chromatograms obtained with SEC-UV-ICP-MS/MS for one replicate of the randomly selected soil from the Swiss soil collection used as quality control. The panels show a) selenium (Se) mass flow (µg L^{-1}), and b) iron (Fe), c) UV, d) sulfur (S), e) copper (Cu), and f) arsenic (As) intensity (counts s^{-1}) chromatograms. The background color indicates the five SEC fractions, i.e., F1 (organo)mineral nanoparticles; F2 larger, more negatively charged, Fe-enriched, aromatic organic matter (OM); F3 smaller, less negatively charged, aromatic OM; F4 small hydrophilic OM; F5 free oxyanions. The vertical dashed lines show the apex of the identified Se fraction peak(s). Please note that the free oxyanions (F5, sharp peak with more or less separated shoulder peaks) are eluting before the small hydrophilic OM (F4, several smaller peaks).

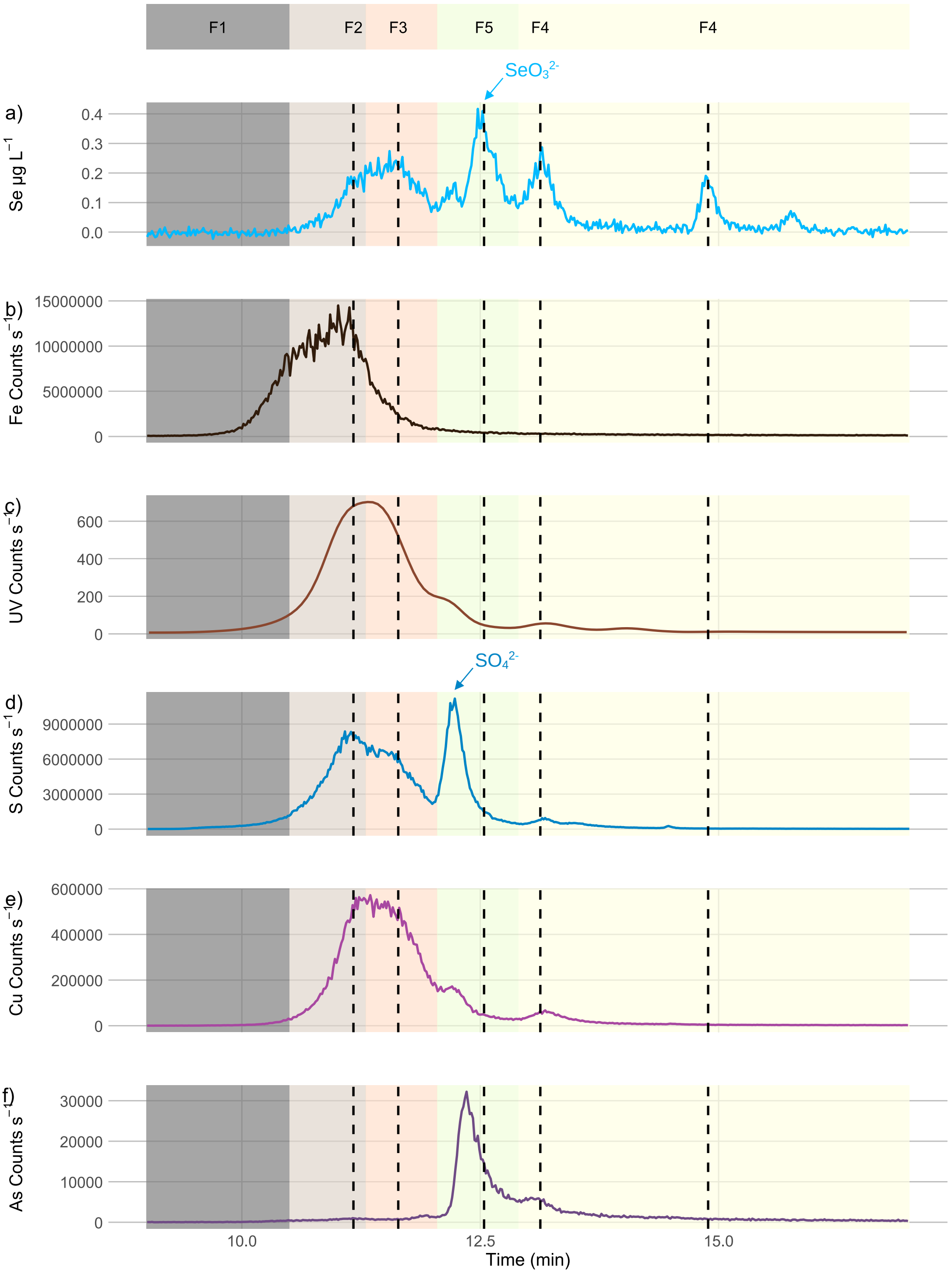

Method S6. Organic matter molecular composition by Py-GC/MS

Organic matter (OM) molecular composition was determined using a multi-shot EGA/PY-3030D Pyrolyzer (Frontier Laboratories Ltd) coupled to a TRACE 1300 GC/ISQ 7000 MS (Thermo Fisher Scientific), as optimized by Tolu et al. (2015).[10] To analyze the same amount of soil organic carbon (SOC) for each sample, 120-1700 µg of soil was weighted using an XP6 balance (Mettler Toledo) into an Eco-cup SF (Frontier Laboratories). The Py-GC interface and GC injector temperatures were set to 340 °C and 320 °C, respectively. The carrier gas in the injector was He and a split ratio of 1/16 or 1/8 was used depending on the sample SOC content, which means that 6.3% or 12.5% of the volatilized molecules were injected in the GC/MS. After one minute the gas-saver mode was used with a flow rate of 3 mL min^{-1} to vent away the pyrolysate bleed of the sample remaining in the pyrolyzer oven. The pyrolysate was separated on a DB-5MS capillary column (30 m 0.25 mm i.d., 0.25 mm film thickness; J&W, Agilent Technologies AB, Sweden). For GC separation, temperature was gradually increased from 40 °C by 10 °C min^{-1} to 320 °C, which was held for 3 min. The GC/MS interface was kept at 300 °C. The MS with a quadrupole type analyzer was operated at unit mass resolution and scanned the mass range from m/z 35 to 500 at 3.1 scan s^{-1}. For ionization, 70 eV electron bombardment was used. A data processing pipeline was used to integrate the Py-GC/MS peaks and extract their mass spectra. The data processing pipeline consists of chromatogram smoothing and alignment, background correction and multivariate curve resolution by alternate regression (MCR-AR). Peak identification was then made using the software "NIST MS Search 2" containing the library "NIST/EPA/NIH 2011" and additional spectra from published studies.[10]

Table S9. Organic matter compounds identified by Py-GC/MS

Table S9. Organic compounds identified by Py-GC/MS, their retention time (RT) in seconds, and how they have been classified and grouped.

RT<br>s	Compound	Class	Group
190	Benzene	(Poly)aromatics	Benzene
281	Toluene	(Poly)aromatics	(Alkyl)benzenes C2-9
283	NA	(Poly)aromatics	(Alkyl)benzenes C2-9
392	Dimethylbenzene	(Poly)aromatics	(Alkyl)benzenes C2-9
415	Styrene	(Poly)aromatics	(Alkyl)benzenes C2-9
491	Benzaldehyde	(Poly)aromatics	Benzaldehyde
576	Benzeneacetaldehyde	(Poly)aromatics	Acetyl-benzene
786	1H-Indene, 4,7-dimethyl-	(Poly)aromatics	Polyaromatics
800	2,3-Dihydro-1-indenone	(Poly)aromatics	Polyaromatics
817	Methylnaphthalene	(Poly)aromatics	Polyaromatics
1099	Alkyl-benzene C10	(Poly)aromatics	(Alkyl)benzenes C10-21
1183	Phenanthrene /Anthracene / 9H-Fluorene, 9-methylene	(Poly)aromatics	Polyaromatics
1297	Alkyl-benzene C13	(Poly)aromatics	(Alkyl)benzenes C10-21
1355	Pyrene / Fluoranthene	(Poly)aromatics	Polyaromatics
1433	Retene	(Poly)aromatics	Polyaromatics
1436	Methylpyrene / Methylfluoranthene	(Poly)aromatics	Polyaromatics
1469	Alkyl-benzene C16	(Poly)aromatics	(Alkyl)benzenes C10-21
1522	Alkyl-benzene C17	(Poly)aromatics	(Alkyl)benzenes C10-21
1565	Triphenylene / Chrysene /Benzo[c]phenanthrene	(Poly)aromatics	Polyaromatics
1712	Alkyl-benzene C21	(Poly)aromatics	(Alkyl)benzenes C10-21
1569	Alkan-2-one C23	Alkan-2-ones	Alkan-2-ones C23-33
1662	Alkan-2-one C25	Alkan-2-ones	Alkan-2-ones C23-33
1926	Alkan-2-one C31	Alkan-2-ones	Alkan-2-ones C23-33
2050	Alkan-2-one C33	Alkan-2-ones	Alkan-2-ones C23-33
803	C13:0	*n*-alkanes	C13:0
1041	C16:0	*n*-alkanes	C16-28:0
1111	C17:0	*n*-alkanes	C17-19:0
1177	C18:0	*n*-alkanes	C16-28:0
1239	C19:0	*n*-alkanes	C17-19:0
1299	C20:0	*n*-alkanes	C16-28:0
1357	C21:0	*n*-alkanes	C21-25:0
1411	C22:0	*n*-alkanes	C16-28:0
1464	C23:0	*n*-alkanes	C21-25:0
1514	C24:0	*n*-alkanes	C16-28:0
1563	C25:0	*n*-alkanes	C21-25:0
1655	C27:0	*n*-alkanes	C27-33:0
1702	C28:0	*n*-alkanes	C16-28:0
1740	C29:0	*n*-alkanes	C27-33:0
1821	C31:0	*n*-alkanes	C27-33:0
1912	C33:0	*n*-alkanes	C27-33:0

Table S9. Continuation 1.

RT<br>s	Compound	Class	Group
1036	C16:1	*n*-alkenes	C16-28:1
1172	C18:1	*n*-alkenes	C16-28:1
1236	C19:1	*n*-alkenes	C17-19:1
1296	C20:1	*n*-alkenes	C16-28:1
1353	C21:1	*n*-alkenes	C21-25:1
1408	C22:1	*n*-alkenes	C16-28:1
1461	C23:1	*n*-alkenes	C21-25:1
1512	C24:1	*n*-alkenes	C16-28:1
1560	C25:1	*n*-alkenes	C21-25:1
1608	C26:1	*n*-alkenes	C16-28:1
1654	C27:1	*n*-alkenes	C27:1
1696	C28:1	*n*-alkenes	C16-28:1
155	Methylfuran	Carbohydrates	(Alkyl)furans (Alkyl)furanones
157	NA	Carbohydrates	(Alkyl)furans (Alkyl)furanones
228	2-Cyclopenten-1-one, 3-methyl-	Carbohydrates	Cyclopentene Pentane Pentenone
265	Methylfuran	Carbohydrates	(Alkyl)furans & furanones
310	(2H)-Furan3-one	Carbohydrates	(Alkyl)furans & furanones
328	3-Furaldehyde	Carbohydrates	(Alkyl)furans (Alkyl)furanones
347	2-Furaldehyde	Carbohydrates	(Alkyl)furans (Alkyl)furanones
347	2-Furaldehyde	Carbohydrates	(Alkyl)furans (Alkyl)furanones
369	2-Furanmethanol	Carbohydrates	(Alkyl)furans (Alkyl)furanones
381	5-Methyl-5H-furan-2-one	Carbohydrates	(Alkyl)furans (Alkyl)furanones
401	4-Cyclopentene-1,3-dione	Carbohydrates	Cyclopentene Pentane Pentenone
430	2(5H)-Furanone + Acetylfuran	Carbohydrates	(Alkyl)furans (Alkyl)furanones
431	NA	Carbohydrates	(Alkyl)furans (Alkyl)furanones
436	Methyl-2-furaldehyde	Carbohydrates	(Alkyl)furans (Alkyl)furanones
446	1,2-Cyclopentanedione	Carbohydrates	Cyclopentene Pentane Pentenone
459	5-Methyl-5H-furan-2-one	Carbohydrates	(Alkyl)furans (Alkyl)furanones
487	Methyl-2-furaldehyde	Carbohydrates	(Alkyl)furans (Alkyl)furanones
528	4-hydroxy-5,6-dihydro(2H)-pyran-2-one	Carbohydrates	(Alkyl)pyranones
540	2-Cyclopenten-1-one, 2-hydroxy-3-methyl-	Carbohydrates	Cyclopentene Pentane Pentenone
552	1,2-Cyclopentanedione, 3-methyl-	Carbohydrates	Cyclopentene Pentane Pentenone
574	Dianhydrorhamnose	Carbohydrates	Anhydrosugars
607	5-formylfurfural	Carbohydrates	(Hydroxy)furans
639	3-hydroxy-2-methyl-(4H)-Pyran-4-one	Carbohydrates	(Alkyl)pyranones
640	Levoglucosenone	Carbohydrates	Anhydrosugars
642	Methylbenzofuran	Carbohydrates	Benzofuran
695	Anhydropentose/anhydro-cyclofuranose	Carbohydrates	Anhydrosugars
736	Dihydrobenzofuran	Carbohydrates	Benzofuran
730	1,4:3,6-Dianhydro-α-d-glucopyranose	Carbohydrates	Anhydrosugars
743	5-(Hydroxymethyl)-2-furaldehyde	Carbohydrates	(Hydroxy)furans
774	Anhydropentose/anhydro-cyclofuranose	Carbohydrates	Anhydrosugars
878	Levoglucosan	Carbohydrates	Anhydrosugars
931	Levoglucosan	Carbohydrates	Anhydrosugars
958	Methoxy-3-methylbenzofuran	Carbohydrates	Benzofuran
965	Levoglucosan	Carbohydrates	Anhydrosugars
968	Levoglucosan	Carbohydrates	Anhydrosugars
1040	1,6-Anhydro-α-d-galactofuranose	Carbohydrates	Anhydrosugars

Table S9. Continuation 2.

740	3-Acetamidofuran	Chitin-derived	Chitin-derived
827	3-acetamido-methylfuran	Chitin-derived	Chitin-derived
1129	Prist-1-ene	Chlorophyll	Pristenes
1226	Phytol / phytadiene 1	Chlorophyll	Phytadienes
1244	Phytadiene	Chlorophyll	Phytadienes
1147	Tetradecanoic acid	Carboxylic acids	Carboxylic acids C14-16
1270	Hexadecenoic acid	Carboxylic acids	Carboxylic acids C14-16
1274	Hexadecanoic acid	Carboxylic acids	Carboxylic acids C14-16
1380	NA	Carboxylic acids	Carboxylic acids C18
1389	NA	Carboxylic acids	Carboxylic acids C18
1576	Alkanoic acid methyl ester	Carboxylic acids	Carboxylic acids C20-24
1588	Docosenoic acid	Carboxylic acids	Carboxylic acids C20-24
1593	Docosanoic acid	Carboxylic acids	Carboxylic acids C20-24
1668	Alkanoic acid methyl ester	Carboxylic acids	Carboxylic acids C20-24
1761	Tetradecanoic acid, tetradecyl ester	Carboxylic acids	Carboxylic acids C20-24
616	Guaiacol	Lignin	Guaiacyl lignin oligomers
681	Benzoic acid	Lignin	Guaiacyl lignin oligomers
715	Methylguaiacol	Lignin	Guaiacyl lignin oligomers
790	Ethylguaiacol	Lignin	Guaiacyl lignin oligomers
822	Vinylguaiacol	Lignin	Guaiacyl lignin oligomers
850	Syringol	Lignin	Syringyl lignin oligomers
860	Propenylquaiacol	Lignin	Guaiacyl lignin oligomers
893	Vanillin	Lignin	Guaiacyl lignin oligomers
898	Propenylquaiacol	Lignin	Guaiacyl lignin oligomers
960	Acetoguaiacone	Lignin	Guaiacyl lignin oligomers
990	Guaiacylacetone	Lignin	Guaiacyl lignin oligomers
1016	4-vinyl- syringol	Lignin	Syringyl lignin oligomers
1083	Syringaldehyde	Lignin	Syringyl lignin oligomers
1101	Allylguaiacol	Lignin	Guaiacyl lignin oligomers
1112	Allylsyringol	Lignin	Syringyl lignin oligomers
1132	Acetosyringone	Lignin	Syringyl lignin oligomers
1138	Coniferaldehyde	Lignin	Guaiacyl lignin oligomers
1290	Sinapic aldehyde	Lignin	Syringyl lignin oligomers

Table S9. Continuation 3.

249	Pyrazine	N compounds	(Alkyl)pyrazines
251	Methylpyrrole	N compounds	(Alkyl)pyrroles
258	Pyridine	N compounds	(Alkyl)pyridines
260	Pyridine	N compounds	(Alkyl)pyridines
262	Pyrrole	N compounds	(Alkyl)pyrroles
448	NA	N compounds	(Alkyl)pyrazines
354	Methylhistamine	N compounds	Diketopiperazines
350	Methylpyrrole	N compounds	(Alkyl)pyrroles
361	Methylpyrrole	N compounds	(Alkyl)pyrroles
448	Methoxyfurangs	N compounds	(Alkyl)pyrazines
455	Dimethylpyridine	N compounds	(Alkyl)pyridines
461	1-Methyl-1H-pyrrole-2,5-dione	N compounds	(Alkyl)pyrroles
489	Tetrahydro-3,6-pyridazinedione	N compounds	(Alkyl)pyrazines
521	Pyrrole-2,5-dione	N compounds	(Alkyl)pyrroles
538	2-Formylpyrrole	N compounds	(Alkyl)pyrroles
591	Acetylpyrrole	N compounds	(Alkyl)pyrroles
593	2-Pyridinecarbonitrile	N compounds	(Alkyl)pyridines
668	Benzeneacetonitrile	N compounds	Aromatic N
760	Benzenepropanenitrile	N compounds	Aromatic N
809	Indole	N compounds	(Alkyl)indoles
811	Phenyl-2-propenenitrile	N compounds	Aromatic N
887	Methylindole	N compounds	(Alkyl)indoles
1125	Diketodipyrrole	N compounds	Diketodipyrrole
1156	Diketopiperazine Cyclo(Pro-Gly)	N compounds	Diketopiperazines
1191	Diketopiperazine Cyclo(Leu-Pro)	N compounds	Diketopiperazines
1216	Methyl N-acetyl-d-glucosamide	N compounds	Chitin-derived
1241	alkanenitrile	N compounds	Alkanenitriles
1261	Diketopiperazine Cyclo(Leu-Pro)	N compounds	Diketopiperazines
1272	Diketopiperazine Cyclo(Pro-Pro)	N compounds	Diketopiperazines
1445	Diketopiperazine Cyclo(Pro-Lys-NH3)	N compounds	Diketopiperazines
1573	Alkanenitrile	N compounds	Alkanenitriles
1667	Alkanenitrile	N compounds	Alkanenitriles
503	Phenol	Phenols	(Alkyl)phenols
580	Phenol, 3-methyl-	Phenols	(Alkyl)phenols
599	Acetophenone	Phenols	(Alkyl)phenols
601	Phenol, 4-methyl-	Phenols	(Alkyl)phenols
602	Phenol, 4-methyl-	Phenols	(Alkyl)phenols
689	2,3-Dimethylphenol	Phenols	(Alkyl)phenols
1597	(2-Phenyl-3-[(phenylsulfinyl)methyl]cyclopropyl)benzene	S compounds	S compounds
1730	Cholest-2-ene	Steroids	Steroids
1990	Stigmasta-3,5-dien-7-one	Steroids	Steroids

Figure S4. Py-GC/MS identified peak area compared to SOC and TN

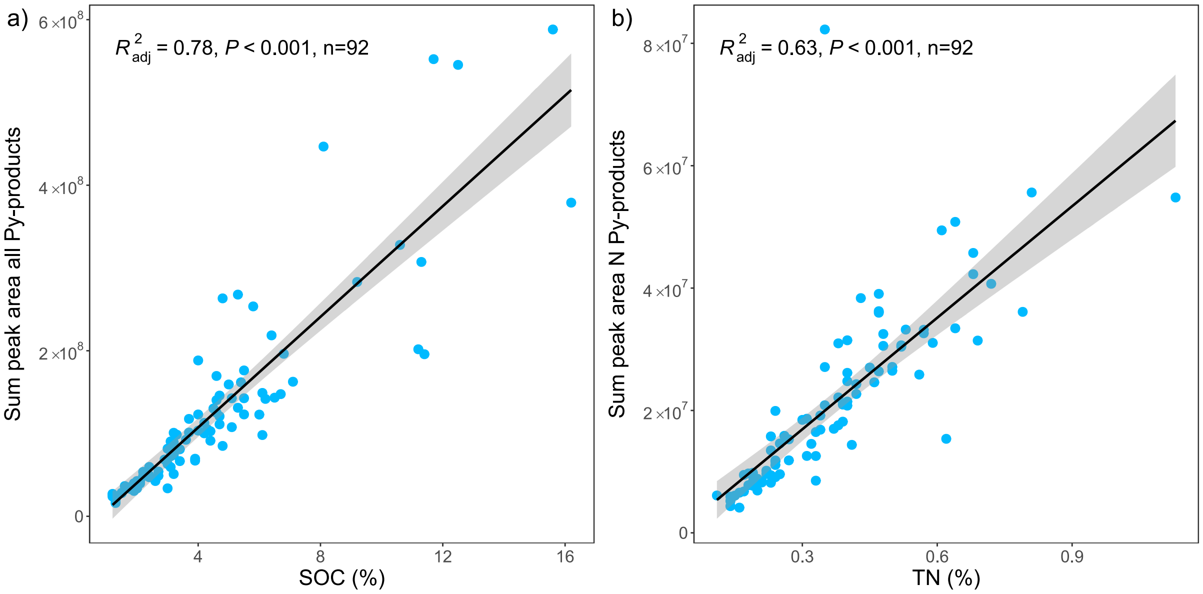

Figure S4. Linear relationships between the sum of Py-GC/MS identified peak areas (signal intensity) of a) all Py-products compared to soil organic carbon (SOC), and b) nitrogen (N) Py-products compared to total N (TN).

Table S10. Analytical reproducibility of Py-GC/MS results per biochemical class

Table S10. Relative standard deviation (RSD) of the relative peak abundance summary statistics (mean, M; standard deviation, SD; minimum, Min; maximum, Max) per organic compound class identified by Py-GC/MS determined across nine samples measured in three analytical replicates.

**Class**	**M RSD** %	**SD RSD** %	**Min RSD** %	**Max RSD** %
(Poly)aromatics	14	11	7	38
Alkan-2-ones	38	29	8	102
n-alkanes	4	2	1	8
n-alkenes	6	4	1	14
Carbohydrates	4	2	1	7
Chitin-derived	8	7	1	26
Chlorophyll	7	3	2	10
Carboxylic acids	25	12	8	42
Lignin	12	5	4	20
N compounds	5	2	2	9
Phenols	6	2	3	9
S compounds	70	22	43	102
Steroids	37	22	2	56

Method S7. Statistical analysis and data visualization

Before assessing differences between land uses, residuals per parameter were tested for normal distribution by the Shapiro-Wilk normality test (*stats* version 4.4.0) and for homogenous variance by the studentized Breusch-Pagan test (*lmtest* version 0.9-40) and the Levene's test (*DescTools* version 0.99.57).[11,12] We further tested for outliers with the Grubbs' test (*outliers* version 0.15).[13] Data manipulation was kept to a minimum to preserve the variability within the data set. Only for assessing land use differences (Table 1, Figure 1), if needed, the data of three converted peat soils (two croplands and one grassland) were removed from the data set as outliers (SOC, TN, pH, S_{Soil} in Table 1 and all Se and S SEC fractions in NaOH extracts in Figure 1) and data were log-transformed (all parameters except pH in Table 1 and Se and S in F2+F3 in Figure 1). Linear models with the response variable as a function of land use were fitted (*stats* version 4.4.0). Pairwise comparisons between land uses were then assessed based on estimated marginal means (*emmeans* version 1.10.5) at 0.05 significance level with Tukey adjustment of P-values for multiple testing.[14] Removal of the three outliers had mostly no influence on the obtained results.

The variability in molecular OM composition was investigated by conducting a principal component analysis (PCA) based on the relative abundances of Py-groups. The data was scaled and centered before computing unrotated PCA following Haaf et al. (2021).[15] The first four PCs together explain 65% of the total variance in OM composition (Figure 2) and were used as indicators of different OM composition aspects in the subsequent analysis.

Correlations between bulk soil properties (SOC, TN, C/N, pH, silt and clay content, ADFE (bulk density), CEC_{pot}, Fe_{oxa}), total element concentrations (Fe_{Soil}, Mn_{Soil}, Ca_{Soil}, Mg_{Soil}), basal soil respiration, MAT and MAP, the first four PC's of the OM data set and Se and S concentrations in soil (Se_{Soil} and S_{Soil}) or Se and S fractions (F2+F3, F4, F5, organic (F2+F3+F4), residual) as proportions in NaOH extracts (in % Se_{NaOH} and % S_{NaOH}) or in soil (in % Se_{Soil} and % S_{Soil}) were calculated based on Spearman's test (*psych* version *2.4.6.26*).[16] Spearman rank correlation coefficients (r_S) and corresponding holm-adjusted P-values are reported in the main text and the Tables S11, S13, and S14.

Figures were produced with *ggplot2* version 3.5.1.[17] When plotting one variable as a function of another, linear models were fitted and the predicted lines and 0.95 confidence intervals, as well as adjusted coefficients of determination (R^2_{adj}), P-values (P), and number of samples (n) of the fitted linear models were computed by *ggpmisc* version 0.6.0.[18] For creating the Swiss map in Figure S1 *sf* version 1.0-16[19,20] and *raster* version 3.6-30[21] were used.

Supplementary Results

Figure S5. SOC, TN, C/N, pH, Se_{Soil} and S_{Soil} per land use

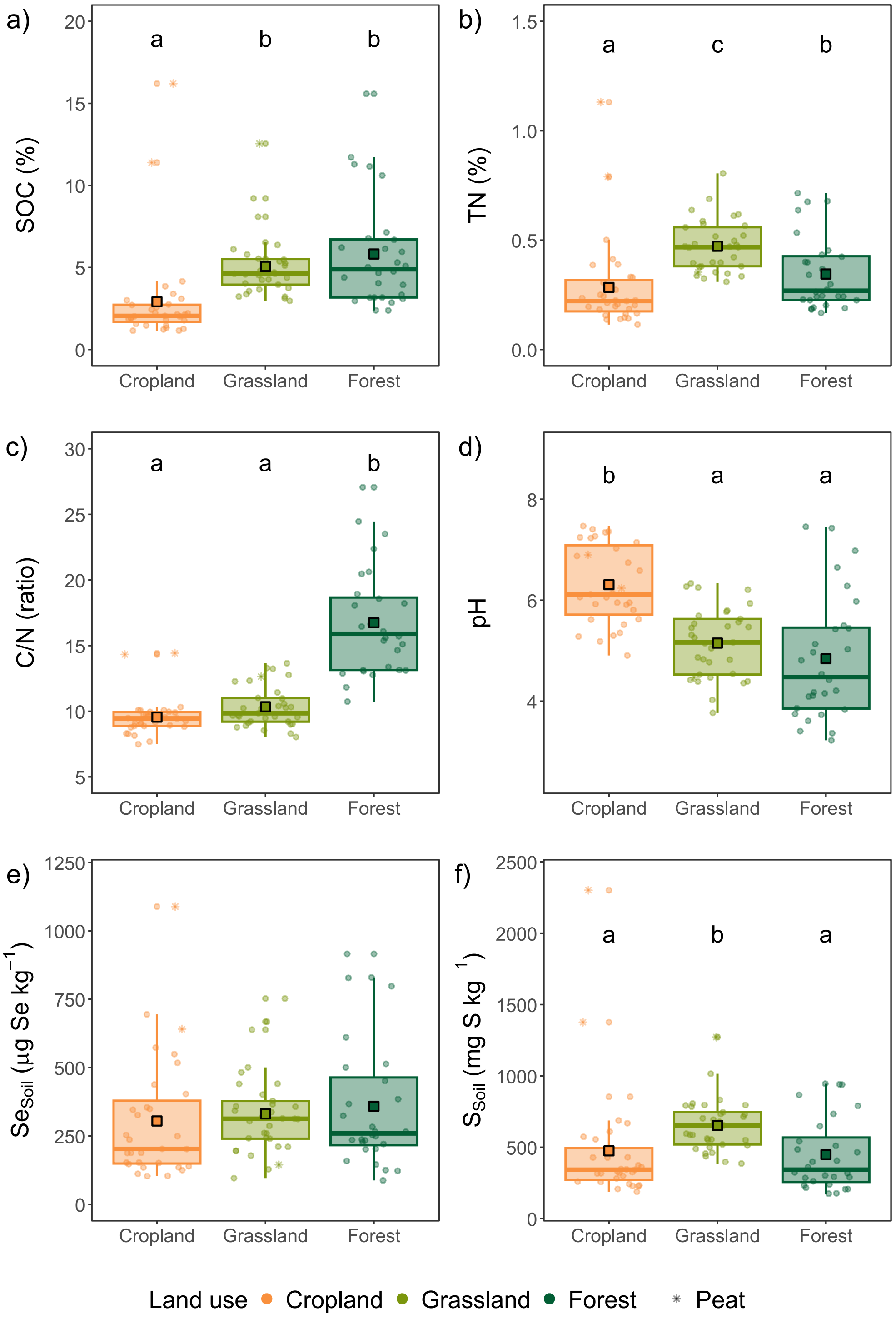

Figure S5. SOC, TN, C/N, pH, and total Se and S concentrations (Se_{Soil} and S_{Soil}) in the Swiss soil collection. The data shows soil organic carbon (SOC, %), total nitrogen (TN, %), C/N (ratio), pH, soil Se (Se_{Soil}, µg Se kg^{-1} soil) and soil S (S_{Soil}, mg S kg^{-1} soil) concentrations. The boxplots show the interquartile range (IQR) as box, representing the middle 50% of the data between the 25th and 75th percentiles, the median marked by the middle line, and the whiskers extending to 1.5 times the IQR. The mean is shown as a square. The points represent the sites. All elements are colored according to land use (cropland, grassland, forest), peat soils are indicated with a star symbol. Different letters denote significant differences between land uses (P≤0.05) based on estimated marginal means.

Table S11. Correlations between soil Se and S concentrations and soil properties

Table S11. Spearman rank correlation coefficients (r_S) and corresponding holm-adjusted P-values between total Se and S concentrations in soil (Se_{Soil} and S_{Soil}) and soil properties, as well as mean annual temperature and precipitation (MAT and MAP). Correlations with r_S>0.5 are highlighted in bold.

**Parameter**	**Se_{Soil}**		**S_{Soil}**	
	r_S	P-value	r_S	P-value
Se_{Soil}	1.00	<0.0001	**0.69**	**<0.0001**
S_{Soil}	**0.69**	**<0.0001**	1.00	<0.0001
SOC	**0.53**	**<0.0001**	**0.74**	**<0.0001**
TN	**0.70**	**<0.0001**	**0.91**	**<0.0001**
C/N	0.02	1.0000	-0.02	1.0000
pH	0.22	0.3269	0.20	0.4784
Silt	0.18	0.5905	0.00	1.0000
Clay	**0.58**	**<0.0001**	0.34	0.0127
ADFE	-0.31	0.0355	-0.49	<0.0001
CEC_{pot}	**0.74**	**<0.0001**	**0.77**	**<0.0001**
Fe_{oxa}	0.45	0.0005	**0.57**	**<0.0001**
Fe_{Soil}	**0.55**	**<0.0001**	0.40	0.0013
Mn_{Soil}	0.11	1.000	0.01	1.000
Ca_{Soil}	0.44	0.0005	0.43	0.0005
Mg_{Soil}	0.27	0.1018	0.34	0.0170
Basal respiration	0.29	0.0659	0.47	0.0001
MAT	-0.11	1.0000	-0.36	0.0069
MAP	0.36	0.0062	0.38	0.0037

Figure S6. NaOH extractabilities of Se, S, and soil organic carbon (SOC)

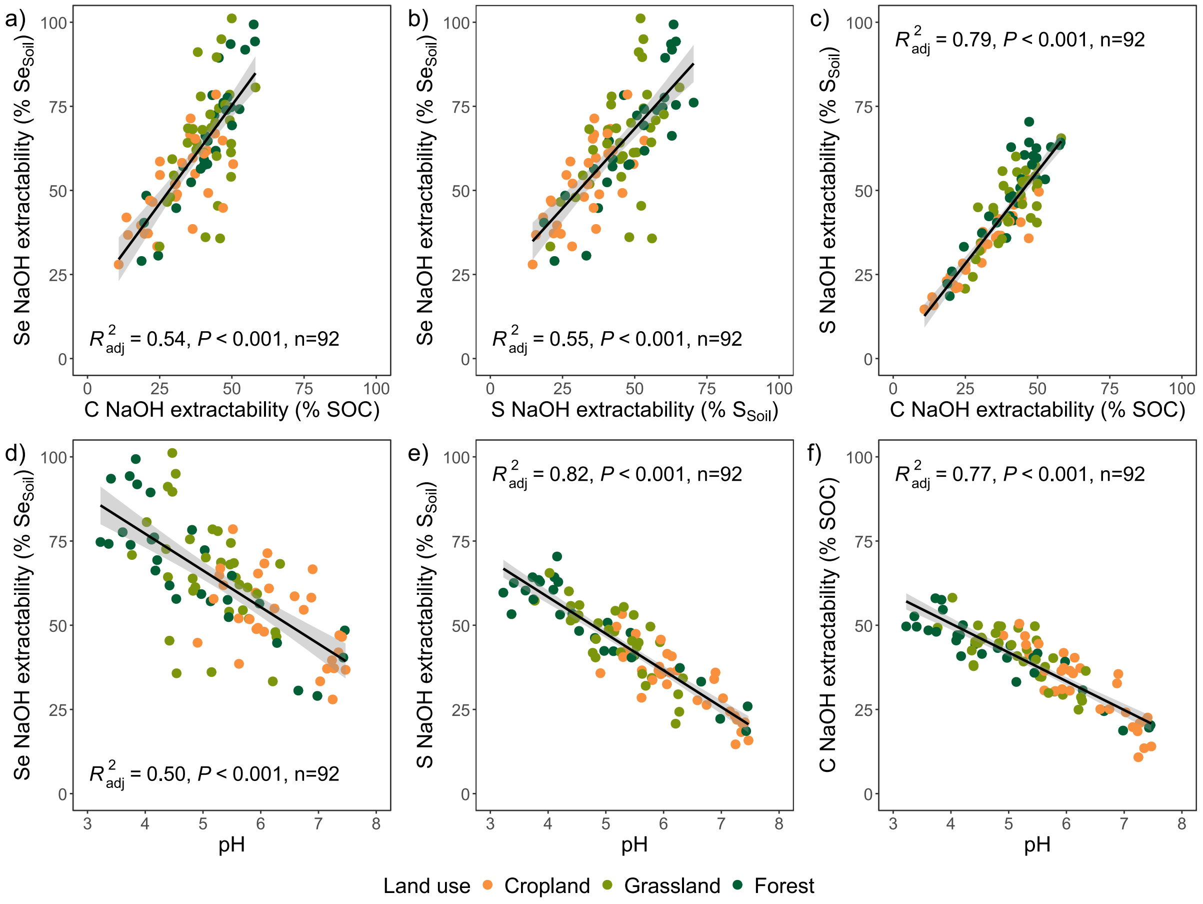

Figure S6. Linear relationships between NaOH extractabilities of a) Se and SOC, b) Se and S, and c) S and SOC, and between NaOH extractabilities of d) Se, e) S, and f) SOC and pH.

Figure S7. Comparison of free Se(IV) concentrations determined by SEC-UV-ICP-MS/MS and AEC-ICP-MS/MS

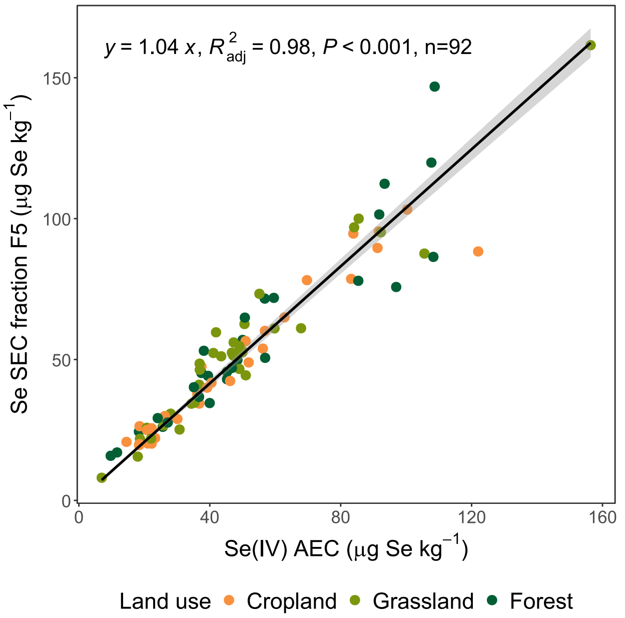

Figure S7. Linear relationship between free Se(IV) concentrations in µg Se kg^{-1} determined by SEC-UV-ICP-MS/MS and AEC-ICP-MS/MS.

Figure S8. Se and S species concentrations in soil per land use

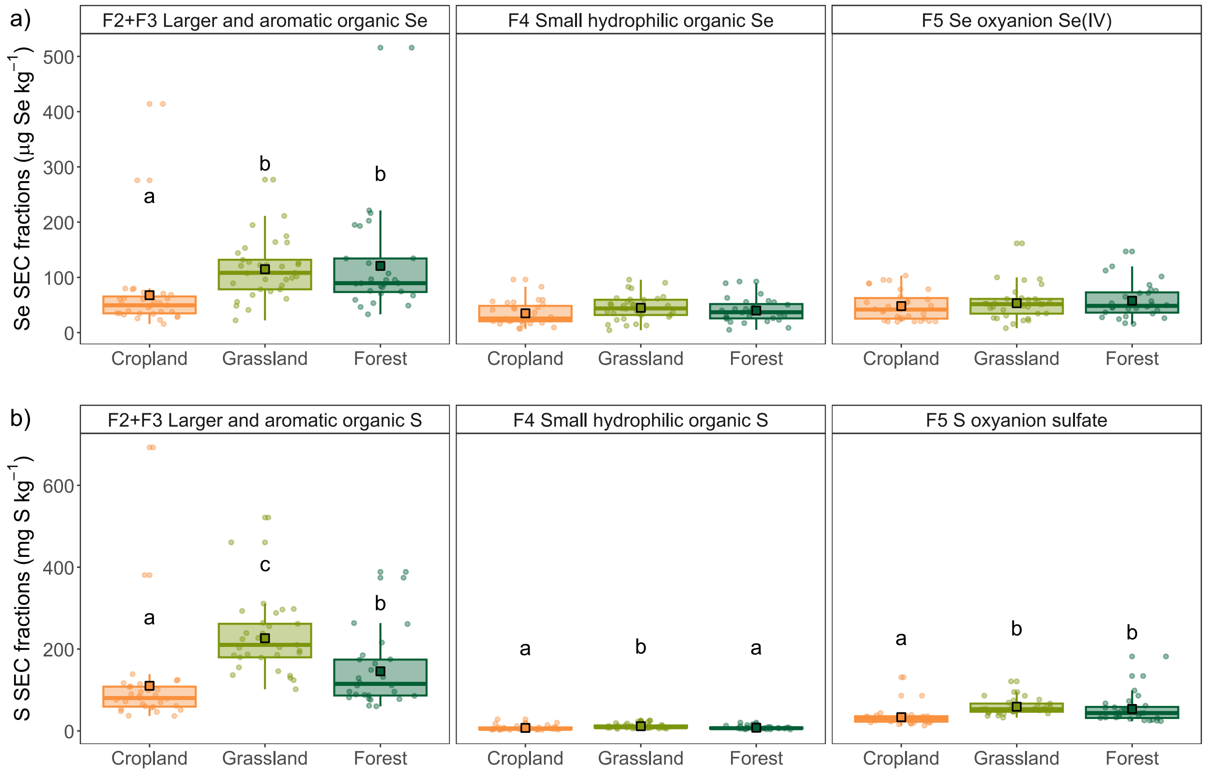

Figure S8. NaOH-extractable Se and S species concentrations in Swiss soils from different land uses determined by SEC-UV-ICP-MS/MS. The data shows the concentrations of Se and S in detected SEC fractions F2+F3 "Se and S associated to larger and aromatic OM", F4 "Se and S associated to small hydrophilic OM", and F5 "Se and S oxyanions" in µg Se kg^{-1} soil and mg S kg^{-1} soil. The boxplots show the interquartile range (IQR) as box, representing the middle 50% of the data between the 25th and 75th percentiles, the median marked by the middle line, and the whiskers extending to 1.5 times the IQR. The mean is shown as a square. The points represent the sites. All elements are colored according to land use (cropland, grassland, forest). Different letters denote significant differences between land uses (P≤0.05) per fraction based on estimated marginal means.

Table S12. Decomposition status assigned to organic matter Py-groups

Table S12. Decomposition status (R = resistant, D = decomposed, F = fresh and/or poorly decomposed, NA = not assigned) assigned to the organic matter Py-groups identified by Py-GC/MS and the biochemical class the Py-groups belong to.

Class	Group	Origin of Py-products	Interpreted decomposition state	References
(Poly)aromatics	Benzene<br>(Alkyl)benzenes C2-9<br>Benzaldehyde<br>Acetyl-benzene<br>Polyaromatics<br>(Alkyl)benzenes C10-21	Degradation products and pyrolysis products of cell wall waxes, biopolymers, and original aromatic plant materials, shown to increase with OM degradation	D	Derenne et al. (1996)[22]<br>Faix et al. (1995)[23]<br>Gelin et al. (1999)[24]<br>Hartgers et al. (1995)[25]<br>Schellekens et al. (2009)[26]
Alkan-2-ones	Alkan-2-ones C23-33	NA	NA	X
Alkanes	C13:0<br>C16-28:0<br>C17-19:0<br>C21-25:0<br>C27-33:0<br>C16-28:1<br>C17-19:1<br>C21-25:1<br>C27:1	Pyrolysis products of fatty acids, cell wall waxes and biopolymers, shown to accumulate with OM degradation	R	Derenne et al. (1996)[22]<br>Gelin et al. (1999)[24]<br>Gupta et al. (2014)[27]<br>Hartgers et al. (1995)[25]<br>Nguyen et al. (2003)[28]<br>Valdés et al. (2013)[29]
Carbohydrates	(Alkyl)furans<br>(Alkyl)furanones<br>Cyclopentene Pentane Pentenone	Pyrolysis products and degradation products of polysaccharides and carbohydrates	D	Buurman et al. (2009)[30]<br>Faix et al. (1995)[23]<br>Nierop et al. (2001)[31]<br>Schellekens et al. (2009)[26]<br>Van Heemst et al. (1996)[32]
	(Alkyl)pyranones<br>Anhydrosugars	Pyrolysis products of polysaccharides, shown to decrease with OM degradation	F	Bindler et al. (2025)[33]<br>Buurman et al. (2009)[30]<br>Kallenbach et al. (2016)[34]<br>Pouwels et al. (1989)[35]<br>Schellekens et al. (2009)[26]
	(Hydroxy)furans<br>Benzofuran	NA	NA	X
Chitin	Chitin-derived	NA	NA	X
Chlorophyll	Pristenes	Pyrolysis and degradation products of chlorophyll (mostly) and tocopherols, shown to accumulate with OM degradation	D, R	Logan et al. (1992)[36]<br>Nguyen et al. (2003)[28]
	Phytadienes	Pyrolysis products of chlorophyll, shown to rapidly decrease with OM degradation	F	Bindler et al. (2025)[33]<br>Ishiwatari et al. (1991)[37]<br>Nguyen et al. (2003)[28]
Fatty acids	Carboxylic acids C14-16<br>Carboxylic acids C18<br>Carboxylic acids C20-24	Pyrolysis products of fatty acids and lipids, shown to decrease with OM degradation	F	Bindler et al. (2025)[33]
Lignin	Guaiacyl lignin oligomers<br>Syringyl lignin oligomers	NA	NA	X

Table S12. Continuation.

Class	Group	Origin of Py-products	Interpreted decomposition state	References
N compounds	(Alkyl)pyrroles<br>(Alkyl)pyridines<br>Diketodipyrrole	Degradation products and pyrolysis products of proteins and amino acids (mostly) as well as of chlorophyll	D	Fabbri et al. (2012)[38]<br>Jokic et al. (2004)[39]<br>Peulvé et al. (1996)[40]<br>Sinninghe Damsté et al. (1992)[41]
	(Alkyl)pyrazines<br>Diketopiperazines	Specific pyrolytic products of intact proteins and amino acids, shown to decrease with OM degradation	F	Bindler et al. (2025)[33]<br>Fabbri et al. (2012)[38]<br>Hendricker and Voorhees (1996)[42]
	(Alkyl)indoles	Pyrolytic products of proteins and amino acids as well as degradation products of proteins and amino acids that are of lower degradation state as compared to the one of (alkyl)pyrroles	Between F & D	Biller et al. (2014)[43]<br>Nguyen et al. (2003)[28]<br>Valdés et al. (2013)[29]
	Aromatic N<br>Alkanenitriles	Pyrolysis products of biomacromolecules (e.g., algeanan) and/or reaction between lipids and proteins during pyrolysis, shown to increase with degradation	D, R	Bindler et al. (2025)[33]<br>Bracewell and Robertson (1984)[44]<br>Gupta et al. (2011)[45]<br>Gupta et al. (2014)[27]<br>Derenne et al. (1996)[22]<br>Peulvé et al. (1996)[40]
Phenols	(Alkyl)phenols	NA	NA	X
S compounds	S compounds	NA	NA	X
Steroids	Steroids	Pyrolysis products of steroids, shown to degrade with OM degradation	F	Bindler et al. (2025)[33]<br>Prost et al. (2018)[46]

Figure S9. Py-groups per land use

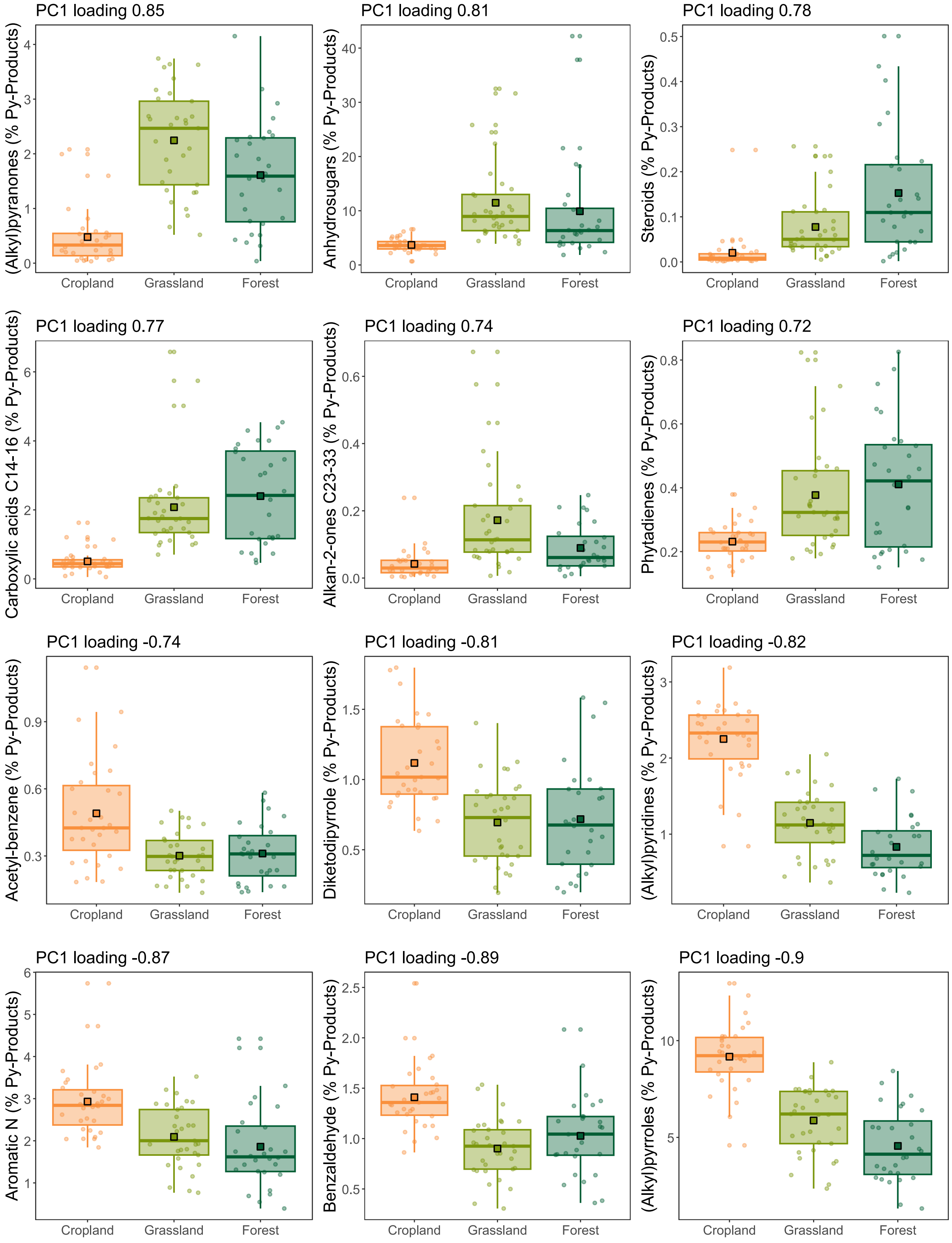

Figure S9. Py-groups per land use. Py-groups with the most positive (top six figures) and most negative (bottom six figures) PC1 loadings are displayed.

Figure S10. Se species as proportions in NaOH extracts as functions of OM PC1 per land use

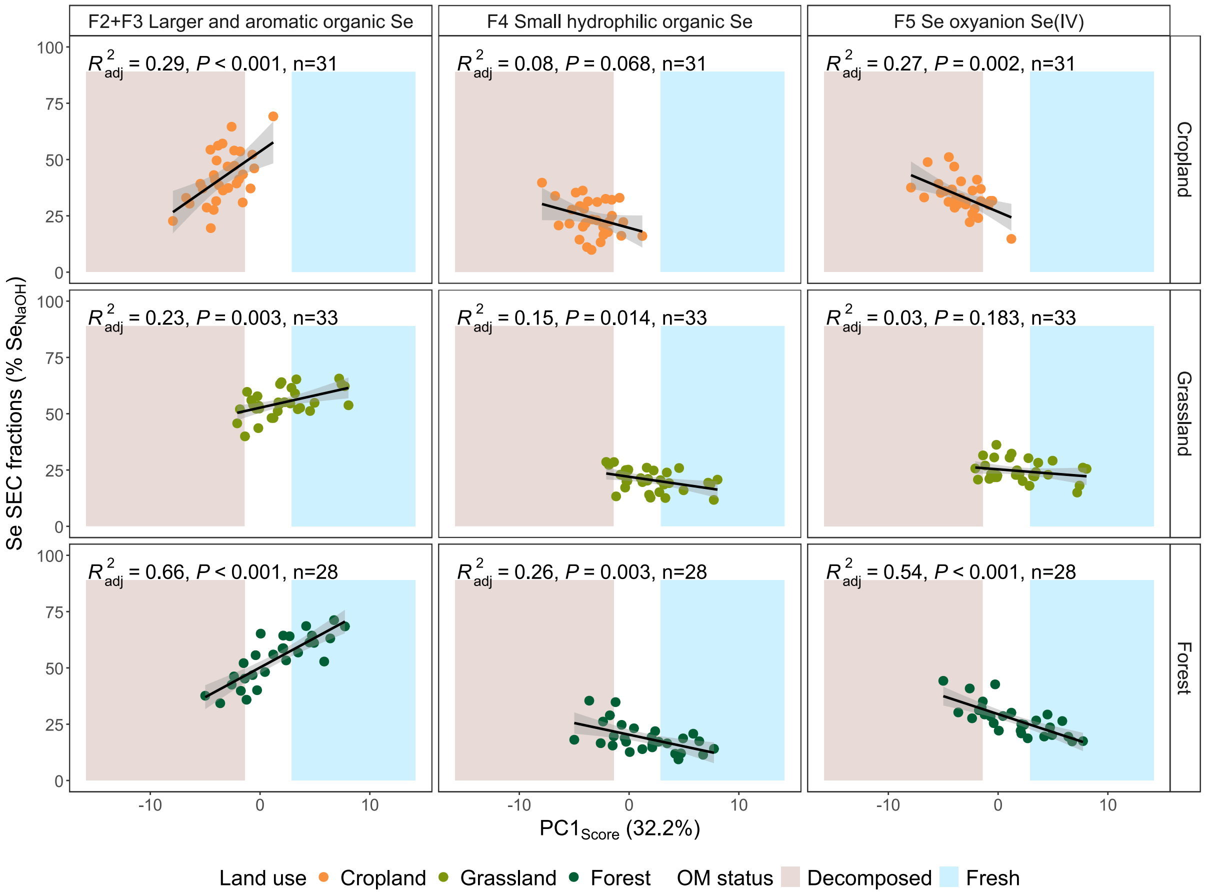

Figure S10. Se fractions (F2+F3, F4, F5) as proportions in NaOH extracts as functions of OM PC1 scores derived from the PCA based on relative abundances of Py-groups for cropland, grassland, and forest. The predicted lines from the linear model fit are shown in black and their 0.95 confidence intervals in grey. The adjusted coefficients of determination (R^2_{adj}), P-values (P), and number of samples (n) of the fitted linear models are reported. The background color indicates the OM decomposition status along PC1 (decomposed versus fresh and/or poorly decomposed). Sites are colored according to land use (cropland, grassland, forest).

Table S13. Correlations between Se and S species as proportions in NaOH extracts and OM PCs and soil properties

Table S13. Spearman rank correlation coefficients (r_S) and corresponding holm-adjusted P-values between a) Se and b) S SEC species (F2+F3, F4, and F5) as proportions in NaOH extracts and OM PCs, soil properties, and MAT and MAP. Correlations with r_S>0.5 are highlighted in bold.

**Parameter**	**F2+F3**		**F4**		**F5**	
	r_S	P-value	r_S	P-value	r_S	P-value
**a) Se SEC fractions (% Se_{NaOH})**						
OM PC1	**0.72**	**<0.0001**	-0.45	0.0002	**-0.69**	**<0.0001**
OM PC2	0.02	1.0000	0.00	1.0000	0.00	1.0000
OM PC3	-0.07	1.0000	0.16	1.0000	-0.04	1.0000
OM PC4	0.00	1.0000	-0.18	1.0000	0.12	1.0000
SOC	**0.52**	**<0.0001**	-0.31	0.0679	**-0.55**	**<0.0001**
TN	0.27	0.2057	-0.04	1.0000	-0.41	0.0027
C/N	**0.52**	**<0.0001**	-0.48	0.0001	-0.40	0.0028
pH	**-0.65**	**<0.0001**	**0.55**	**<0.0001**	**0.53**	**<0.0001**
Silt	-0.37	0.0127	0.21	0.8796	0.35	0.0261
Clay	-0.46	0.0002	0.32	0.0630	0.38	0.0101
ADFE	**-0.55**	**<0.0001**	0.39	0.0055	**0.51**	**<0.0001**
CEC_{pot}	0.16	1.0000	-0.02	1.0000	-0.29	0.1779
Fe_{oxa}	0.43	0.0027	-0.33	0.0698	-0.37	0.0252
Fe_{Soil}	-0.19	1.0000	0.11	1.0000	0.16	1.0000
Mn_{Soil}	-0.33	0.0372	0.18	1.0000	0.37	0.0119
Ca_{Soil}	-0.49	0.0001	0.49	0.0001	0.28	0.2028
Mg_{Soil}	-0.23	0.6007	0.33	0.0542	0.04	1.0000
Basal respiration	0.34	0.0328	-0.16	1.0000	-0.41	0.0027
MAT	-0.48	0.0001	0.30	0.1121	0.47	0.0001
MAP	0.27	0.2037	-0.16	1.0000	-0.27	0.2121
**b) S SEC fractions (% S_{NaOH})**						
OM PC1	0.40	0.0042	-0.35	0.0333	-0.33	0.0614
OM PC2	-0.10	1.0000	0.19	1.0000	0.02	1.0000
OM PC3	-0.03	1.0000	0.16	1.0000	-0.04	1.0000
OM PC4	-0.40	0.0047	0.07	1.0000	0.43	0.0012
SOC	0.36	0.0182	-0.28	0.2857	-0.32	0.0787
TN	0.44	0.0006	-0.20	1.0000	-0.44	0.0006
C/N	0.00	1.0000	-0.19	1.0000	0.07	1.0000
pH	-0.34	0.0379	0.41	0.0030	0.24	0.8035
Silt	-0.08	1.0000	0.10	1.0000	0.05	1.0000
Clay	-0.19	1.0000	0.09	1.0000	0.19	1.0000
ADFE	-0.28	0.2897	0.29	0.1934	0.22	1.0000
CEC_{pot}	0.16	1.0000	-0.19	1.0000	-0.10	1.0000
Fe_{oxa}	0.46	0.0009	-0.30	0.2857	-0.43	0.0035
Fe_{Soil}	0.03	1.0000	-0.01	1.0000	-0.01	1.0000
Mn_{Soil}	-0.06	1.0000	0.13	1.0000	0.04	1.0000
Ca_{Soil}	-0.26	0.5375	0.28	0.3010	0.21	1.0000
Mg_{Soil}	-0.06	1.0000	0.03	1.0000	0.08	1.0000
Basal respiration	0.15	1.0000	-0.14	1.0000	-0.11	1.0000
MAT	-0.38	0.0109	0.32	0.0787	0.33	0.0617
MAP	0.33	0.0558	-0.36	0.0217	-0.26	0.4277

Figure S11. Se and S species as proportions in NaOH extracts as functions of pH

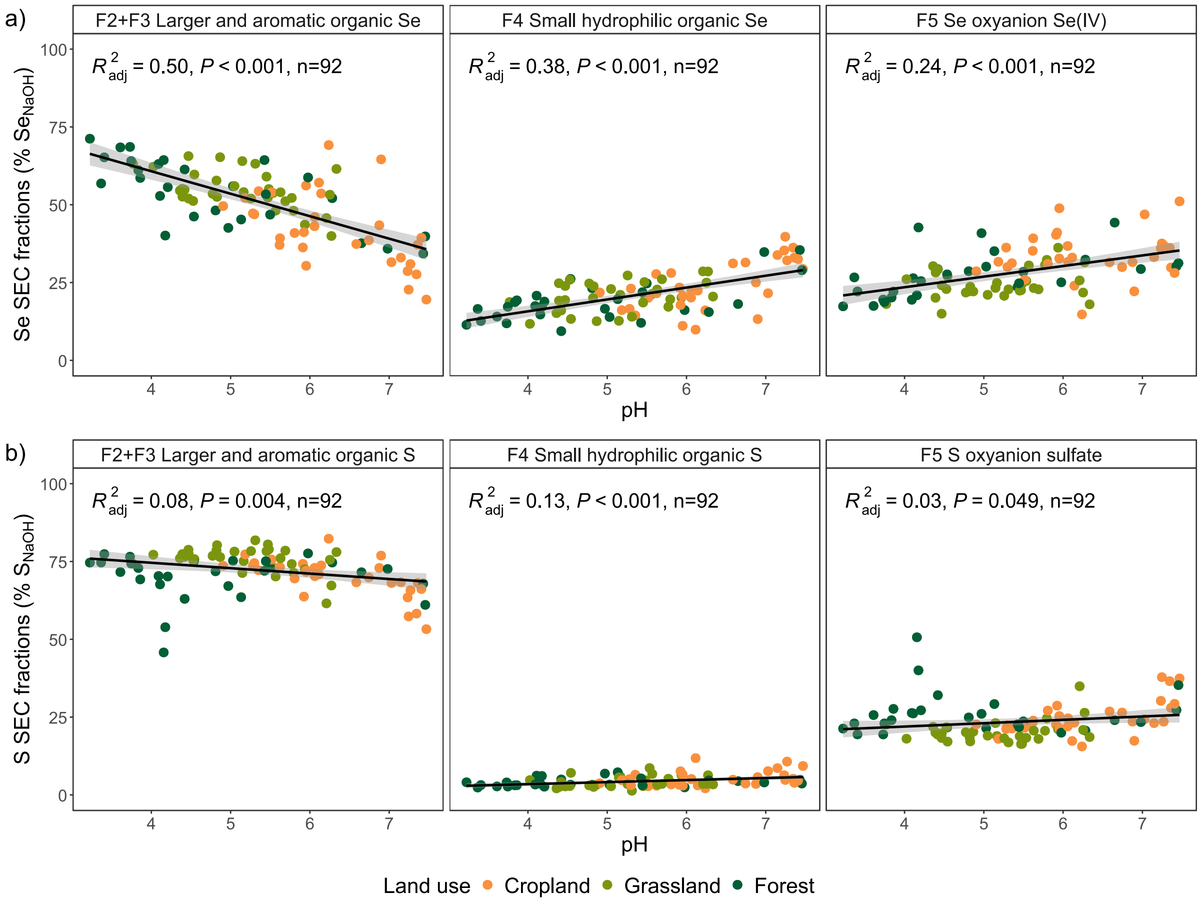

Figure S11. a) Se and b) S fractions (F2+F3, F4, F5) as proportions in NaOH extracts as functions of pH. The predicted lines from the linear model fit are shown in black and their 0.95 confidence intervals in grey. The adjusted coefficients of determination (R^2_{adj}), P-values (P), and number of samples (n) of the fitted linear models are reported. Sites are colored according to land use (cropland, grassland, forest).

Figue S12: Residual Se and S

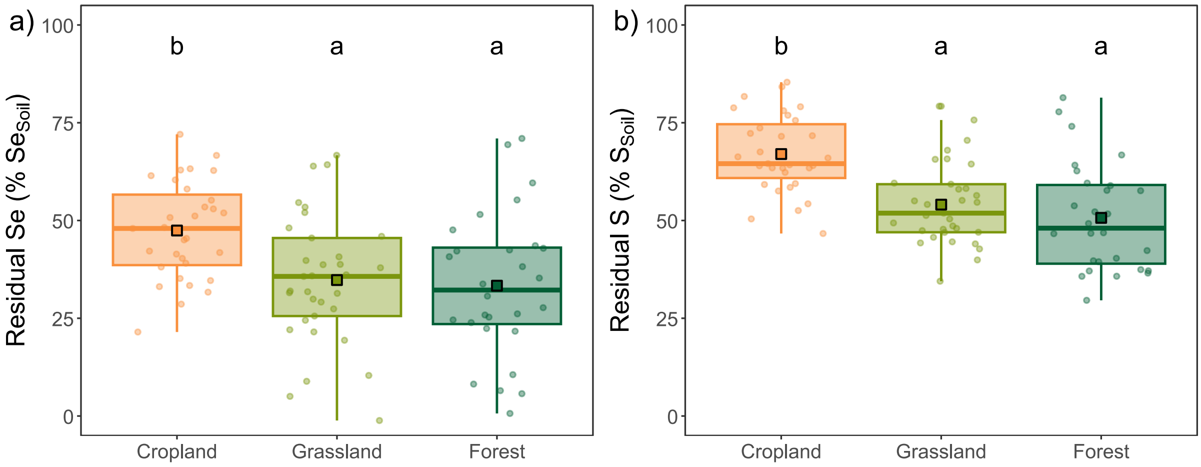

Figure S12. Residual Se and S nonextractable by NaOH of Swiss soils from different land uses. The data shows the proportions of a) Se and b) S nonextractable by NaOH in % of total Se and S (i.e., Se_{Soil} and S_{Soil}). The boxplots show the interquartile range (IQR) as box, representing the middle 50% of the data between the 25th and 75th percentiles, the median marked by the middle line, and the whiskers extending to 1.5 times the IQR. The mean is shown as a square. The points represent the sites. All elements are colored according to land use (cropland, grassland, forest). Different letters denote significant differences between land uses ($P \leq 0.05$) based on estimated marginal means.

Table S14. Correlations between Se and S species as proportions in soil and OM PCs and soil properties

Table S14. Spearman rank correlation coefficients (r_S) and corresponding holm-adjusted P-values between a) Se or b) S SEC species (F2+F3, F4, and F5, organic (F2+F3+F4) and residual) as proportions in soil and OM PCs, soil properties, and MAT and MAP. Correlations with r_S>0.5 are highlighted in bold.

**Parameter**	**F2+F3**		**F4**		**F5**		**Organic (F2+F3+F4)**		**Residual**	
	r_S	P-value	r_S	P-value	r_S	P-value	r_S	P-value	r_S	P-value
**a) Se SEC fractions (% Se_{Soil})**										
OM PC1	**0.73**	**<0.0001**	0.08	1.0000	-0.01	1.0000	**0.68**	**<0.0001**	**-0.60**	**<0.0001**
OM PC2	0.01	1.0000	0.01	1.0000	0.02	1.0000	0.00	1.0000	0.00	1.0000
OM PC3	-0.21	1.0000	-0.09	1.0000	-0.35	0.0367	-0.21	1.0000	0.31	0.1613
OM PC4	-0.02	1.0000	-0.19	1.0000	0.06	1.0000	-0.05	1.0000	0.01	1.0000
SOC	0.38	0.0142	-0.19	1.0000	-0.38	0.0123	0.30	0.1960	-0.17	1.0000
TN	0.15	1.0000	-0.10	1.0000	-0.43	0.0014	0.13	1.0000	0.01	1.0000
C/N	0.44	0.0010	-0.20	1.0000	-0.07	1.0000	0.35	0.0418	-0.29	0.3073
pH	**-0.74**	**<0.0001**	-0.07	1.0000	-0.28	0.3734	**-0.70**	**<0.0001**	**0.70**	**<0.0001**
Silt	-0.32	0.1157	-0.01	1.0000	0.11	1.0000	-0.30	0.2712	0.24	1.0000
Clay	-0.46	0.0005	-0.11	1.0000	-0.10	1.0000	-0.45	0.0009	0.44	0.0014
ADFE	-0.39	0.0092	0.26	0.6157	0.34	0.0510	-0.29	0.3108	0.17	1.0000
CEC_{pot}	0.06	1.0000	-0.14	1.0000	-0.40	0.0096	0.04	1.0000	0.09	1.0000
Fe_{oxa}	0.36	0.0575	-0.17	1.0000	-0.16	1.0000	0.30	0.3723	-0.22	1.0000
Fe_{Soil}	-0.37	0.0238	-0.30	0.2085	-0.35	0.0430	-0.40	0.0064	0.46	0.0003
Mn_{Soil}	-0.46	0.0004	-0.22	1.0000	-0.16	1.0000	-0.48	0.0001	0.47	0.0002
Ca_{Soil}	**-0.57**	**<0.0001**	-0.02	1.0000	-0.39	<0.0001	**-0.53**	**<0.0001**	**0.60**	**<0.0001**
Mg_{Soil}	-0.29	0.3348	0.07	1.0000	-0.29	0.3430	-0.25	0.9586	0.32	0.1621
Basal resp.	0.23	1.0000	-0.14	1.0000	-0.39	0.0097	0.18	1.0000	-0.07	1.0000
MAT	-0.38	0.0120	0.08	1.0000	0.21	1.0000	-0.33	0.0946	0.23	1.0000
MAP	0.41	0.0034	0.13	1.0000	0.19	1.0000	0.41	0.0035	-0.41	0.0035
**b) S SEC fractions (% S_{Soil})**										
OM PC1	**0.73**	**<0.0001**	0.25	0.6794	**0.56**	**<0.0001**	**0.72**	**<0.0001**	**-0.70**	**<0.0001**
OM PC2	0.00	1.0000	0.13	1.0000	-0.05	1.0000	0.01	1.0000	-0.03	1.0000
OM PC3	-0.37	0.0170	-0.16	1.0000	-0.40	0.0054	-0.38	0.0161	0.39	0.0085
OM PC4	-0.06	1.0000	0.07	1.0000	0.22	1.0000	-0.06	1.0000	-0.04	1.0000
SOC	0.36	0.0277	0.00	1.0000	0.16	1.0000	0.34	0.0515	-0.31	0.1318
TN	0.13	1.0000	-0.15	1.0000	-0.19	1.0000	0.11	1.0000	-0.03	1.0000
C/N	0.41	0.0033	0.18	1.0000	**0.51**	**<0.0001**	0.41	0.0034	-0.47	0.0002
pH	**-0.88**	**<0.0001**	-0.39	0.0088	**-0.83**	**<0.0001**	**-0.88**	**<0.0001**	**0.90**	**<0.0001**
Silt	-0.24	0.9147	-0.10	1.0000	-0.22	1.0000	-0.24	0.9427	0.24	0.8983
Clay	-0.39	0.0114	-0.20	1.0000	-0.33	0.0955	-0.39	0.0091	0.38	0.0178
ADFE	-0.38	0.0129	-0.02	1.0000	-0.28	0.3708	-0.37	0.0205	0.35	0.0359
CEC_{pot}	-0.02	1.0000	-0.24	0.9437	-0.15	1.0000	-0.04	1.0000	0.06	1.0000
Fe_{oxa}	0.41	0.0118	0.06	1.0000	0.14	1.0000	0.38	0.0249	-0.36	0.0554
Fe_{Soil}	-0.24	0.9427	-0.22	1.0000	-0.30	0.2104	-0.25	0.6794	0.25	0.6794
Mn_{Soil}	-0.28	0.3913	-0.11	1.0000	-0.27	0.4562	-0.28	0.3708	0.31	0.1665
Ca_{Soil}	**-0.75**	**<0.0001**	-0.42	0.0044	**-0.74**	**<0.0001**	**-0.76**	**<0.0001**	**0.79**	**<0.0001**
Mg_{Soil}	-0.31	0.1845	-0.28	0.4517	-0.35	0.0515	-0.33	0.1132	0.36	0.0457
Basal resp.	0.23	1.0000	0.03	1.0000	0.15	1.0000	0.22	1.0000	-0.22	1.0000
MAT	-0.49	0.0001	-0.07	1.0000	-0.31	0.1318	-0.48	0.0001	0.44	0.0009
MAP	0.36	0.0265	0.01	1.0000	0.24	0.9147	0.35	0.0385	-0.35	0.0431

Figure S13. Organic Se proportions in soil as a function of SOC for the Swiss soil collection and the Hawaii soils studied by Tolu et al. (2022)

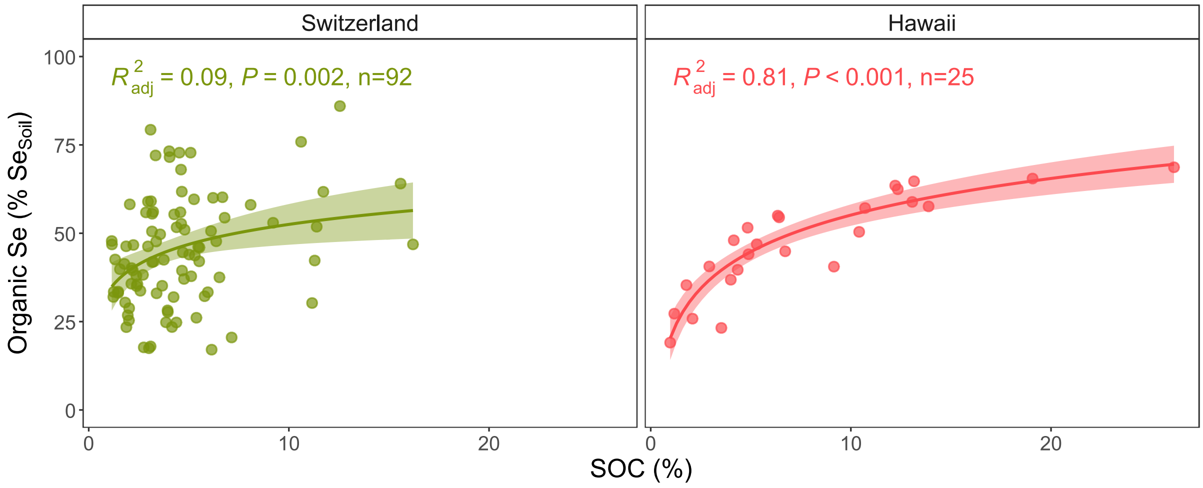

Figure S13. The proportion of NaOH-extractable organic Se (F2+F3+F4) in the analyzed collection of Swiss soils and in the Hawaii soils studied by Tolu et al. (2022)[8] as a log-function of soil organic C (SOC).

Figure S14. S F2+F3 and residual S as proportions in soil as functions of organic matter PC1 and pH per land use

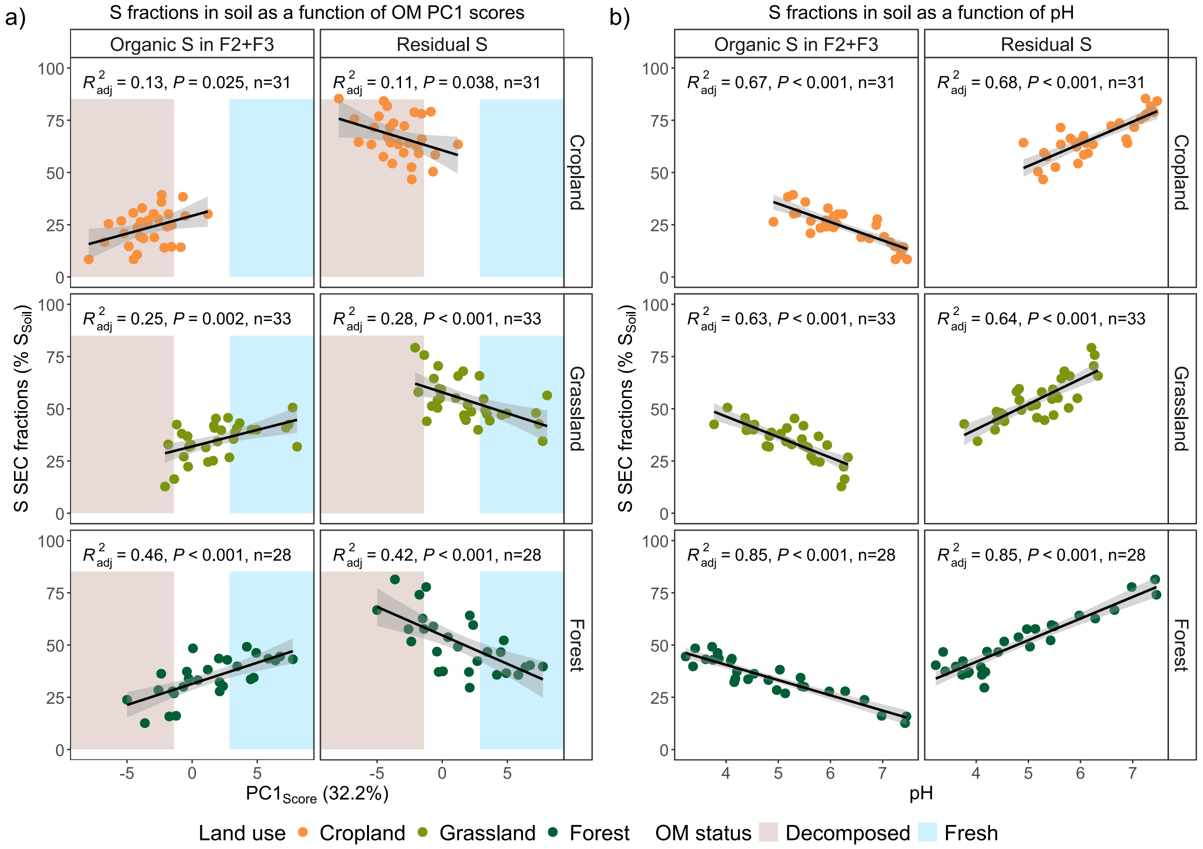

Figure S14. Larger and aromatic S F2+F3 and residual S as proportions in soil as functions of a) OM PC1 scores derived from the PCA based on relative abundances of Py-groups, and b) pH per land use. The predicted lines from the linear model fit are shown in black and their 0.95 confidence intervals in grey. The adjusted coefficients of determination (R^2_{adj}), P-values (P), and number of samples (n) of the fitted linear models are reported. The background color in a) indicates the OM decomposition status along PC1 (decomposed versus fresh and/or poorly decomposed). Sites are colored according to land use (cropland, grassland, forest).

References

(1) Moll-Mielewczik, J.; Keel, S. G.; Gubler, A. Organic Carbon Contents of Mineral Grassland Soils in Switzerland over the Last 30 Years. *Agric. Ecosyst. Environ.* **2023**, *342*, 108258. https://doi.org/10.1016/j.agee.2022.108258.

(2) Gubler, A.; Wächter, D.; Schwab, P.; Müller, M.; Keller, A. Twenty-Five Years of Observations of Soil Organic Carbon in Swiss Croplands Showing Stability Overall but with Some Divergent Trends. *Environ. Monit. Assess.* **2019**, *191* (5), 277. https://doi.org/10.1007/s10661-019-7435-y.

(3) FAL. *Schweizerische Referenzmethoden Der Eidg. Landwirtschaftlichen Forschungsanstalten*; Zürich-Reckenholz, 1996.

(4) Hug, A.-S.; Gubler, A.; Gschwend, F.; Widmer, F.; Oberholzer, H.-R.; Frey, B.; Meuli, R. G. NABObio – Bodenbiologie in Der Nationalen Boden Beobachtung Ergebnisse 2012–2016 Handlungsempfehlungen Und Indikatoren. *Agroscope Sci.* **2018**, *63*.

(5) Schwab, P.; Gubler, A. Methoden Zur Bestimmung Physikalischer Begleitparameter an Bodenproben. *Agroscope Sci.* **2016**, *40*, 1–34.

(6) Reusser, J. E.; Siegenthaler, M. B.; Winkel, L. H. E.; Wächter, D.; Kretzschmar, R.; Meuli, R. G. *Geochemical Soil Atlas of Switzerland: Distribution of 20 Elements in the Topsoil*; Agroscope: Zurich, 2023. https://doi.org/10.34776/gca23-e.

(7) Tolu, J.; Le Hécho, I.; Bueno, M.; Thiry, Y.; Potin-Gautier, M. Selenium Speciation Analysis at Trace Level in Soils. *Anal. Chim. Acta* **2011**, *684* (1), 126–133. https://doi.org/10.1016/j.aca.2010.10.044.

(8) Tolu, J.; Bouchet, S.; Helfenstein, J.; Hausheer, O.; Chékifi, S.; Frossard, E.; Tamburini, F.; Chadwick, O. A.; Winkel, L. H. E. Understanding Soil Selenium Accumulation and Bioavailability through Size Resolved and Elemental Characterization of Soil Extracts. *Nat. Commun.* **2022**, *13* (1), 6974. https://doi.org/10.1038/s41467-022-34731-6.

(9) Laborda, F.; Jiménez-Lamana, J.; Bolea, E.; Castillo, J. R. Selective Identification, Characterization and Determination of Dissolved Silver(i) and Silver Nanoparticles Based on Single Particle Detection by Inductively Coupled Plasma Mass Spectrometry. *J. Anal. At. Spectrom.* **2011**, *26* (7), 1362–1371. https://doi.org/10.1039/C0JA00098A.

(10) Tolu, J.; Gerber, L.; Boily, J.-F.; Bindler, R. High-Throughput Characterization of Sediment Organic Matter by Pyrolysis–Gas Chromatography/Mass Spectrometry and Multivariate Curve Resolution: A Promising Analytical Tool in (Paleo)Limnology. *Anal. Chim. Acta* **2015**, *880*, 93–102. https://doi.org/10.1016/j.aca.2015.03.043.

(11) Zeileis, A.; Hothorn, T. Diagnostic Checking in Regression Relationships. *R News* **2002**, *2* (3), 7–10.

(12) Signorell, A. *DescTools: Tools for Descriptive Statistics. R package version 0.99.57*. https://CRAN.R-project.org/package=DescTools (accessed 2024-10-07).

(13) Komsta, L. *Outliers: Tests for outliers. R package version 0.14*.

(14) Lenth, R. V. *emmeans: Estimated Marginal Means, aka Least-Squares Means. R package version 1.10.5*. https://rvlenth.github.io/emmeans/ (accessed 2024-10-31).

(15) Haaf, D.; Six, J.; Doetterl, S. Global Patterns of Geo-Ecological Controls on the Response of Soil Respiration to Warming. *Nat. Clim. Change* **2021**, *11* (7), 623–627. https://doi.org/10.1038/s41558-021-01068-9.

(16) Revelle, W. *psych: Procedures for Psychological, Psychometric, and Personality Research*. https://CRAN.R-project.org/package=psych.

(17) Hadley Wickham. *Ggplot2: Elegant Graphics for Data Analysis*; Springer-Verlag: New York, 2016.

(18) Aphalo, P. *ggpmisc: Miscellaneous Extensions to "ggplot2". R package version 0.6.0*. https://github.com/aphalo/ggpmisc, https://docs.r4photobiology.info/ggpmisc/.

(19) Pebesma, E. Simple Features for R: Standardized Support for Spatial Vector Data. *R J.* **2018**, *10* (1), 439. https://doi.org/10.32614/rj-2018-009.

(20) Pebesma, E.; Bivand, R. *Spatial Data Science: With Applications in R*, 1st ed.; Chapman and Hall/CRC: New York, 2023. https://doi.org/10.1201/9780429459016.

(21) Hijmans, R. J. *raster: Geographic Data Analysis and Modeling. R package version 3.6-30*. https://CRAN.R-project.org/package=raster (accessed 2025-07-20).

(22) Derenne, S.; Largeau, C.; Berkaloff, C. First Example of an Algaenan Yielding an Aromatic-Rich Pyrolysate. Possible Geochemical Implications on Marine Kerogen Formation. *Org. Geochem.* **1996**, *24* (6), 617–627. https://doi.org/10.1016/0146-6380(96)00053-8.

(23) Faix, O.; Fortmann, I.; Bremer, J.; Meier, D. Thermal Degradation Products of Wood. *Holz Als Roh-Werkst.* **1991**, *49* (5), 213–219. https://doi.org/10.1007/BF02613278.

(24) Gelin, F.; Volkman, J. K.; Largeau, C.; Derenne, S.; Sinninghe Damsté, J. S.; De Leeuw, J. W. Distribution of Aliphatic, Nonhydrolyzable Biopolymers in Marine Microalgae. *Org. Geochem.* **1999**, *30* (2), 147–159. https://doi.org/10.1016/S0146-6380(98)00206-X.

(25) Hartgers, W. A.; Sinninghe Damsté, J. S.; de Leeuw, J. W. Curie-Point Pyrolysis of Sodium Salts of Functionalized Fatty Acids. *J. Anal. Appl. Pyrolysis* **1995**, *34* (2), 191–217. https://doi.org/10.1016/0165-2370(94)00881-Z.

(26) Schellekens, J.; Buurman, P.; Pontevedra-Pombal, X. Selecting Parameters for the Environmental Interpretation of Peat Molecular Chemistry – A Pyrolysis-GC/MS Study. *Org. Geochem.* **2009**, *40* (6), 678–691. https://doi.org/10.1016/j.orggeochem.2009.03.006.

(27) Gupta, N. S.; Steele, A.; Fogel, M.; Griffin, P.; Adams, M.; Summons, R. E.; Yang, H.; Cody, G. D. Experimental Formation of Geomacromolecules from Microbial Lipids. *Org. Geochem.* **2014**, *67*, 35–40. https://doi.org/10.1016/j.orggeochem.2013.11.006.

(28) Nguyen, R. T.; Harvey, H. R.; Zang, X.; van Heemst, J. D. H.; Hetényi, M.; Hatcher, P. G. Preservation of Algaenan and Proteinaceous Material during the Oxic Decay of Botryococcus Braunii as Revealed by Pyrolysis-Gas Chromatography/Mass Spectrometry and 13C NMR Spectroscopy. *Org. Geochem.* **2003**, *34* (4), 483–497. https://doi.org/10.1016/S0146-6380(02)00261-9.

(29) Valdés, F.; Catalá, L.; Hernández, M. R.; García-Quesada, J. C.; Marcilla, A. Thermogravimetry and Py–GC/MS Techniques as Fast Qualitative Methods for Comparing the Biochemical Composition of Nannochloropsis Oculata Samples Obtained under Different Culture Conditions. *Bioresour. Technol.* **2013**, *131*, 86–93. https://doi.org/10.1016/j.biortech.2012.12.133.

(30) Buurman, P.; Nierop, K. G. J.; Kaal, J.; Senesi, N. Analytical Pyrolysis and Thermally Assisted Hydrolysis and Methylation of EUROSOIL Humic Acid Samples — A Key to Their Source. *Geoderma* **2009**, *150* (1), 10–22. https://doi.org/10.1016/j.geoderma.2008.12.012.

(31) Nierop, K. G. J.; van Lagen, B.; Buurman, P. Composition of Plant Tissues and Soil Organic Matter in the First Stages of a Vegetation Succession. *Geoderma* **2001**, *100* (1), 1–24. https://doi.org/10.1016/S0016-7061(00)00078-1.

(32) Van Heemst, J. D. H.; Peulve, S.; De Leeuw, J. W. Novel Algal Polyphenolic Biomacromolecules as Significant Contributors to Resistant Fractions of Marine Dissolved and Particulate Organic Matter. *Org. Geochem.* **1996**, *24* (6), 629–640. https://doi.org/10.1016/0146-6380(96)00054-X.

(33) Bindler, R.; Tolu, J.; Bigler, C.; Rydberg, J.; Martínez Cortizas, A. Carbon Burial (in)Efficiency: Tracking the Molecular Fingerprint of In Situ Organic Matter Burial Using a 30-Year Freeze-Core Series From a Northern Boreal Lake (Nylandssjön, Sweden). *J. Geophys. Res. Biogeosciences* **2025**, *130* (3), e2024JG008397. https://doi.org/10.1029/2024JG008397.

(34) Kallenbach, C. M.; Frey, S. D.; Grandy, A. S. Direct Evidence for Microbial-Derived Soil Organic Matter Formation and Its Ecophysiological Controls. *Nat. Commun.* **2016**, *7* (1), 13630. https://doi.org/10.1038/ncomms13630.

(35) Pouwels, A. D.; Eijkel, G. B.; Boon, J. J. Curie-Point Pyrolysis-Capillary Gas Chromatography-High-Resolution Mass Spectrometry of Microcrystalline Cellulose. *J. Anal. Appl. Pyrolysis* **1989**, *14* (4), 237–280. https://doi.org/10.1016/0165-2370(89)80003-8.

(36) Logan, G. A.; Boon, J. J.; Eglinton, G. Structural Biopolymer Preservation in Miocene Leaf Fossils from the Clarkia Site, Northern Idaho. *Proc. Natl. Acad. Sci.* **1993**, *90* (6), 2246–2250. https://doi.org/10.1073/pnas.90.6.2246.

(37) Ishiwatari, M.; Ishiwatari, R.; Sakashita, H.; Tatsumi, T.; Tominaga, H. Pyrolysis of Chlorophyll a after Preliminary Heating at a Moderate Temperature: Implications for the Origin of Prist-1-Ene on Kerogen Pyrolysis. *J. Anal. Appl. Pyrolysis* **1991**, *18* (3), 207–218. https://doi.org/10.1016/0165-2370(91)87002-4.

(38) Fabbri, D.; Adamiano, A.; Falini, G.; De Marco, R.; Mancini, I. Analytical Pyrolysis of Dipeptides Containing Proline and Amino Acids with Polar Side Chains. Novel 2,5-Diketopiperazine Markers in the Pyrolysates of Proteins. *J. Anal. Appl. Pyrolysis* **2012**, *95*, 145–155. https://doi.org/10.1016/j.jaap.2012.02.001.

(39) Jokic, A.; Schulten, H.-R.; Cutler, J. N.; Schnitzer, M.; Huang, P. M. A Significant Abiotic Pathway for the Formation of Unknown Nitrogen in Nature. *Geophys. Res. Lett.* **2004**, *31* (5). https://doi.org/10.1029/2003GL018520.

(40) Peulvé, S.; Leeuw, J. W. de; Sicre, M.-A.; Baas, M.; Saliot, A. Characterization of Macromolecular Organic Matter in Sediment Traps from the Northwestern Mediterranean Sea. *Geochim. Cosmochim. Acta* **1996**, *60* (7), 1239–1259. https://doi.org/10.1016/0016-7037(95)00442-4.

(41) Sinninghe Damsté, J. S.; Xavier, F.; Heras, C. de las; Leeuw, J. W. de. Molecular Analysis of Sulphur-Rich Brown Coals by Flash Pyrolysis—Gas Chromatography—Mass Spectrometry: The Type III-S Kerogen. *J. Chromatogr. A* **1992**, *607* (2), 361–376. https://doi.org/10.1016/0021-9673(92)87096-Q.

(42) Hendricker, A. D.; Voorhees, K. J. An Investigation into the Curie-Point Pyrolysis-Mass Spectrometry of Glycyl Dipeptides. *J. Anal. Appl. Pyrolysis* **1996**, *36* (1), 51–70. https://doi.org/10.1016/0165-2370(95)00920-5.

(43) Biller, P.; Ross, A. B. Pyrolysis GC–MS as a Novel Analysis Technique to Determine the Biochemical Composition of Microalgae. *Algal Res.* **2014**, *6*, 91–97. https://doi.org/10.1016/j.algal.2014.09.009.

(44) Bracewell, J. M.; Robertson, G. W. Quantitative Comparison of the Nitrogen-Containing Pyrolysis Products and Amino Acid Composition of Soil Humic Acids. *J. Anal. Appl. Pyrolysis* **1984**, *6* (1), 19–29. https://doi.org/10.1016/0165-2370(84)80002-9.

(45) Gupta, N. S.; Cody, G. D. Identification and Characterization of Chitin in Organisms. In *Chitin: Formation and Diagenesis*; Gupta, N. S., Ed.; Springer Netherlands: Dordrecht, 2011; pp 117–132. https://doi.org/10.1007/978-90-481-9684-5_6.

(46) Prost, K.; Bradel, P. L.; Lehndorff, E.; Amelung, W. Steroid Dissipation and Formation in the Course of Farmyard Manure Composting. *Org. Geochem.* **2018**, *118*, 47–57. https://doi.org/10.1016/j.orggeochem.2017.12.006.
